# Machine Learning-Driven Discovery of Indole/Oxoindole–Piperazine Scaffolds as Dual MAO-B/Sig-1R Ligands for Neurodegenerative Disorders

**DOI:** 10.1021/acs.jcim.6c00379

**Published:** 2026-07-07

**Authors:** Ulisses Candido da Silva, Miller Santos Ferreira, Eduardo Borba Alves, Valéria Vieira Moura Paixão, Igor Rodrigues Lapa, Micaela M. Barbosa Nogueira, Ricardo Pereira Rodrigues, Wanda Pereira Almeida, Marisi Gomes Soares, Daniela A. Chagas-Paula, Albert Katchborian-Neto, Tiago Branquinho Oliveira, Danielle Ferreira Dias

**Affiliations:** † Institute of Chemistry, Federal University of Alfenas (UNIFAL), Alfenas, Minas Gerais 37130-00, Brazil; ‡ Department of Pharmacy, Federal University of Sergipe (UFS), São Cristóvão, Sergipe 49107-230, Brazil; § Department of Chemistry, Federal University of Juiz de Fora, Juizde Fora, Minas Gerais 36036-900, Brazil; ∥ Department of Chemistry, Federal University of São Carlos (UFSCAR), São Carlos, São Paulo 13565-905, Brazil; ⊥ School of Pharmaceutical Sciences, State University of Campinas (UNICAMP), Campinas, São Paulo 13083-871, Brazil

## Abstract

The monoamine oxidase B (MAO-B) enzyme and Sigma-1 (Sig-1R) receptor are therapeutically relevant targets implicated in neurodegenerative and neuropsychiatric disorders. Dual modulation of these proteins represents an attractive multitarget strategy for central nervous system drug discovery. In this study, we focused on indole/oxindole-based scaffolds, including indolamide and oxindole cores with piperazine motifs (often *N*-aryl piperazines), as a working hypothesis for multitarget MAO-B/Sig-1R modulation. A ligand-based machine learning approach was applied to build robust quantitative structure–activity relationship (QSAR) classifiers capable of identifying potential modulators of both targets. Using curated ChEMBL data sets, support vector machine models were trained with molecular descriptors selected via L1 regularization and forward feature selection. The final models achieved strong predictive performance (κ > 0.78; accuracy >88%) and revealed key structural determinants of activity, including aromatic bridging/planarity, polarizability-related terms, and complementary donor–acceptor patterns. Virtual screening of 147 natural compounds using the developed QSAR models, followed by applicability domain filtering, identified 11 compounds with predicted dual-target potential. Subsequent docking-based analysis prioritized four compounds with plausible predicted binding modes at both targets, while molecular dynamics simulations (150 ns) supported the short-time scale stability of 2 selected protein–ligand complexes. Overall, this work establishes general SAR principles for dual MAO-B/Sig-1R modulation and highlights how descriptor-driven modeling can inform molecular design and the prioritization of compounds for synthesis in multitarget medicinal chemistry programs.

## Graphical abstract

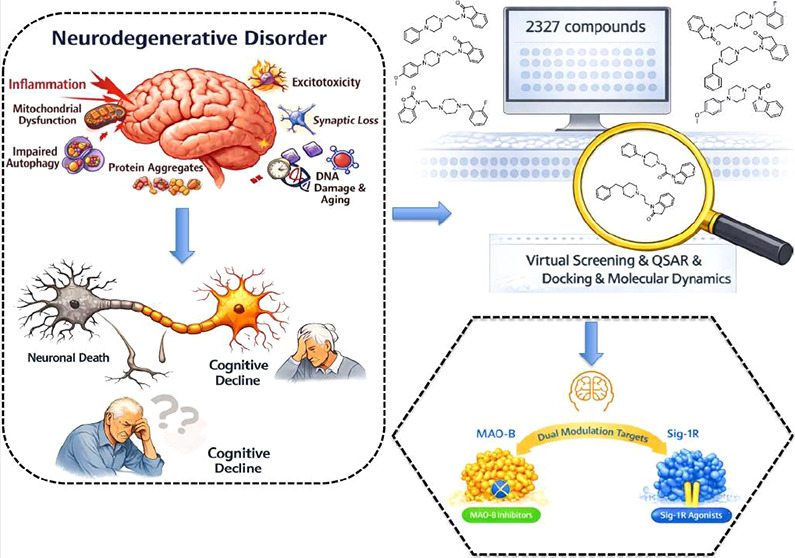

## 1. Introduction

Neurodegenerative and neuropsychiatric disorders, including Parkinson’s disease, Alzheimer’s disease, and resistant depressive disorder, represent an escalating global health crisis. These complex conditions share overlapping pathophysiological mechanisms, including oxidative stress, mitochondrial dysfunction, neuroinflammation, and disrupted monoaminergic signaling pathways.^1–5^ These shared features underscore the limitations of single-target therapies and highlight the need for more integrative treatment strategies. In recent years, multitarget-directed ligands (MTDLs) have emerged as promising therapeutic candidates capable of engaging multiple nodes within interconnected neurobiological pathways. By simultaneously modulating complementary mechanisms, MTDLs may improve therapeutic efficacy and reduce side effects. This is particularly relevant in central nervous system (CNS) disorders, where the biological interplay of neurotransmission and cellular stress responses contributes to both symptom progression and treatment resistance.^6,7^

Two molecular targets of particular interest in this context are monoamine oxidase B (MAO-B) and the Sigma-1 receptor (Sig-1R). MAO-B is a mitochondrial flavoenzyme involved in the catabolism of monoaminergic neurotransmitters, and its upregulation with age and disease progression contributes to neurodegeneration through the generation of oxidative byproducts.^3,6,8,9^ Clinically, MAO-B inhibitors like selegiline and rasagiline are used in Parkinson’s disease management, reinforcing the pharmacological relevance of this target.^6,7^ In parallel, Sig-1R is an atypical intracellular chaperone located at the endoplasmic reticulum–mitochondria interface. It regulates key stress-adaptive processes, including calcium homeostasis, mitochondrial function, and inflammatory signaling, which are mechanisms directly tied to neuronal survival.^10–13^ The availability of high-resolution Sig-1R crystal structures has clarified the physicochemical demands of its hydrophobic β-barrel binding pocket, supporting rational ligand design.^4,14,15^

Furthermore, traditional high-throughput screening approaches are resource-intensive and limited in their ability to probe large, chemically diverse libraries. In contrast, computational strategies offer scalable and efficient alternatives during early drug discovery stages.^14,16^ Quantitative structure–activity relationship (QSAR) models allow for the identification of molecular descriptors correlated with biological activity, enabling rational prioritization of compound scaffolds before synthesis or experimental testing. When complemented by structure-based methods such as molecular docking and molecular dynamics simulations, these models provide deeper insight into ligand-target interactions, predicted binding modes, and complex stability, key features for multitarget drug design involving CNS targets such as MAO-B and Sig-1R.^14,15^

The current study aims to discover and prioritize potential dual-acting ligands that simultaneously target MAO-B and Sig-1R using a multilevel computational approach. By screening a curated library of natural and nature-inspired compounds, this work combines QSAR modeling, molecular docking, and molecular dynamics simulations to prioritize novel multitarget neuroprotective candidates. By integrating ligand-based and structure-based computational approaches, this work seeks to provide a rational framework for the discovery of multitarget candidates and to advance the design and synthesis of novel MAO-B/Sig-1R modulators with applicability for CNS disorders.

## 2. Materials and Methods

### 2.1 Database Acquisition

Data for building the MAO-B inhibition models (IC_50_) were obtained from ChEMBL (CHEMBL2039). In total, 3270 compounds tested in *Homo sapiens* under the MAO-B “single protein” assay format were retained after filtering. In addition, Sig-1R binding affinity data (*K_i_*) were collected from ChEMBL (CHEMBL287), yielding 827 *H. sapiens* compounds under the same assay format. Both data sets were then curated to retain only entries with IC_50_ (MAO-B) or *K_i_* (Sig-1R) values reported in nM. SMILES were converted to 2D structures and curated by salt removal, fragment cleaning ([N+]­(=O)­[O−] e [S]­(=O)­(=O)), and element filtering (C, H, O, N, S, F, and Cl). Structures were then protonated (hydrogens added), converted to 3D, charges standardized, and aromatized, followed by energy minimization using MMFF94s in KNIME and a final optimization with MOPAC/PM7. IC_50_ and *K_i_* values were converted to mol·L^–1^ to compute pIC_50_ (−log IC_50_) and p*K_i_* (−log *K_i_*). MAO-B compounds were labeled as active when pIC_50_ ≥ 5.47 (median) and inactive otherwise, whereas Sig-1R compounds were considered active when p*K_i_* ≥ 7.^16,17^ To improve class separability, compounds with activity values close to the decision boundary were excluded using KNIME-based filters (0.009 for MAO-B and 0.016 for Sig-1R), and the final data sets were class-balanced using the Equal Size Sampling node.^18,19^ Importantly, the binary classification strategy was used as an operational prioritization framework rather than as an absolute definition of binding or nonbinding behavior. Because affinity and inhibitory potency are continuous variables, compounds located very close to the decision threshold may carry higher labeling uncertainty. To mitigate this issue, an activity buffer zone was applied during data curation, and compounds with activity values close to the cutoff were excluded before model training. Thus, the final classification data sets were enriched in compounds with clearer activity–class separation, reducing the likelihood that nearly equivalent pIC_50_ or p*K_i_* values would be assigned to opposite classes. Furthermore, a virtual screening library comprising 147 literature-reported natural and nature-inspired compounds was assembled for external prioritization. These molecules were selected based on their structural compatibility with the indole/oxindole–piperazine chemical space investigated in this study, their potential synthetic accessibility, and the absence, to the best of our knowledge, of previous experimental evaluation against both MAO-B and Sig-1R targets. The structures were standardized and curated using the same preprocessing workflow applied to the training data sets, ensuring consistency in descriptor calculation, applicability domain assessment, QSAR prediction, and subsequent docking analyses. These structures were curated and preprocessed using the same workflow adopted for the training data sets, ensuring consistency in descriptor calculation, applicability domain assessment, QSAR prediction, and subsequent docking analyses.

### 2.2 Molecular Descriptor Calculation and Construction of QSAR Models

Molecular descriptors for the MAO-B and Sig-1R data sets were calculated using the AlvaDesc v2.0 software. The same structural preprocessing applied to the data sets was also used for the virtual screening set to ensure consistency in descriptor calculation. The MAO-B and Sig-1R data sets were split 80:20 into training and external test sets. Training descriptors were filtered by low variance (threshold = 0.05) and high pairwise correlation (|*r*| ≥ 0.97) to reduce multicollinearity, and the training data were then MIN–MAX normalized to the 0.1–1 range. The external test set and the virtual screening data set were normalized using the training set parameters. Descriptor selection was performed via L1-regularized logistic regression (LASSO), retaining only features with nonzero coefficients (sign indicates direction of association with the active class; magnitude indicates contribution strength). The retained descriptors were further refined using forward feature selection, which iteratively adds variables to maximize performance.^20–22^ For MAO-B, forward selection was carried out with an SVM (SVM Learner) using a polynomial kernel, whereas for Sig-1R, a logistic regression-based classifier was used. The number of selected descriptors was limited to 13–15, and the selection procedure was embedded in a 10-fold cross-validation (*k* = 10) to reduce overfitting. Permutation importance was computed by permuting each descriptor in the training set while keeping the others unchanged; a larger performance drop indicates a more relevant descriptor for separating active and inactive compounds.^23^ Final classification models were implemented in KNIME and evaluated using sensitivity, specificity, precision, F1 score, and accuracy, then applied to virtual screening to identify candidates with potential MAO-B inhibition or Sig-1R binding affinity.

In addition, exploratory regression analyses were performed by using the descriptors selected in the classification workflow to evaluate their suitability for continuous activity modeling. However, these preliminary models did not achieve sufficiently robust predictive performance and were, therefore, not included in the main screening workflow.

### 2.3 Applicability Domain for Virtual Screening Sets

The applicability domain (APD) for the MAO-B and Sig-1 virtual screening data sets was calculated using the descriptors selected in the developed models. Structures falling within the APD were subsequently evaluated using the SVM Learner model. The APD was determined using the “Domain Similarity” node,^24^ which automatically applies the equation: APD = *d̅* + *Z*σ. This equation was applied throughout the workflow to evaluate groups of structures and determine whether each compound fell within the chemical space represented by the training set. Here, *d̅* represents the mean Euclidean distance of the training set, σ is the corresponding standard deviation, and *Z* is an empirical parameter set to 0.5. A compound was considered within the applicability domain when its APD value was lower than the threshold defined for the training set.

### 2.4 Docking for MAO-B and Sig-1R

Crystallographic structures of the targets were retrieved from the Protein Data Bank (PDB) based on the availability of suitable ligand–protein complexes.^25^ MAO-B (PDB ID: 2 V5Z) and the Sig-1R receptor (PDB ID: 5HK1) were selected because they contain cocrystallized ligands and provide biologically relevant binding-site information. Protein and ligand structures were prepared in the PDBQT format^26^ (required for AutoDock Vina). Preparation included the removal of crystallographic water molecules, the addition of polar hydrogens, and the assignment of Gasteiger partial charges. Ligands were assigned appropriate protonation states at physiological pH, and rotatable bonds were defined. The docking search space was defined to fully encompass each binding site, with grid boxes centered on the reference ligand coordinates: for MAO-B, *x* = 51.886, *y* = 156.453, *z* = 28.559, and for Sig-1R, *x* = 11.755, *y* = 37.191, *z* = −34.868, using identical box dimensions (25 Å^3^). Docking calculations were carried out with AutoDock Vina using an exhaustiveness value of 128, which controls the thoroughness of conformational sampling. Up to 10 poses per ligand (num_modes = 10) were generated and ranked according to the Vina scoring function.^27^ The docking protocol was validated by redocking the reference ligands and, additionally, by cross-docking crystallographic ligands from independent PDB complexes into the receptor structures used in the main study, followed by RMSD analysis and visual comparison between predicted and crystallographic poses (Supporting Information, Tables S13–S16 and Figures S21–S24). Predicted binding affinities (kcal·mol^–1^) were used for comparative ranking within each data set (not as absolute free energies), and binding modes were inspected for key interactions (hydrophobic contacts, π–π interactions, hydrogen bonds) and ligand proximity to cofactors or catalytically relevant regions. Finally, all 147 ligands from the virtual screening set were docked against both targets, considering only the best pose per ligand (lowest predicted affinity) to enable a consistent assessment of multitarget versus selective binding behavior.

### 2.5 Molecular Dynamics for MAO-B and Sig-1R

Compounds predicted to be active against both MAO-B and Sig-1R and exhibiting the best docking scores were selected for molecular dynamics (MD) simulations using GROMACS v2023.3. Protein topologies were generated with the OPLS-AA/M force field, and protein–ligand complexes were built in a cubic simulation box with the protein centered and a minimum distance of 1 nm from the box edges.^28^ Systems were solvated with TIP3P water, and ligand/cofactor topologies were generated using ACPYPE and MKTOP.^29–31^ All systems were neutralized with Na^+^ and Cl^–^ ions (0.15 mol·L^–1^), energy-minimized using the steepest descent algorithm (maximum force <1000 kJ·mol^–1^·nm^–1^), and equilibrated for 1 ns under NVT and NPT ensembles using V-rescale and Berendsen thermostats (310 K, 1 bar), followed by 150 ns production runs.^32,33^ These simulations were employed as a short-term structural stability filter to verify the maintenance of docking-predicted binding modes under dynamic conditions, rather than to assess long-term kinetic parameters or global conformational changes. Trajectories were analyzed using PyMOL and VMD, and structural clustering was performed with gmx cluster (GROMOS algorithm, 0.2 nm cutoff) based on Cα RMSD after system stabilization to obtain representative conformations and ensure convergence.^34^

## 3. Results and Discussion

### 3.1 Theoretical Basis and Mechanistic Rationale for Target Selection

The design of MTDLs has emerged as a superior paradigm for treating the multifactorial nature of neurodegeneration.^35,36^ Nature-inspired compounds represent a reservoir of chemical diversity and evolutionary-refined biological relevance for CNS drug discovery. Their structural complexity and evolutionary adaptation for protein interaction make them promising candidates for MTDL strategies.^14,36,37^ Notably, MAO-B and Sig-1R receptors exhibit a significant pharmacophoric overlap; both targets feasibly accommodate scaffolds featuring aromatic systems, hydrophobic moieties, and essential basic nitrogen groups. Indole and oxindole–piperazine templates, motifs frequently encountered in neuroactive alkaloids, can be exploited due to their shared structural requirements.^38–40^ The developed approach aimed at designing synergistic neuroactive agents capable of concurrently modulating enzymatic oxidative stress (MAO-B) and chaperone-mediated proteostasis (Sig-1R).

In the CNS, MAO-B activity becomes particularly relevant with aging, as MAO-B levels increase, especially in astrocytes of the substantia nigra, contributing to enhanced dopaminergic vulnerability, oxidative stress, and the bioactivation of neurotoxins like MPTP.^6,8^ Clinically, newer agents like safinamide combine MAO-B inhibition with additional mechanisms, such as glutamatergic modulation, highlighting the translational relevance of this target across both neurodegenerative and neuropsychiatric conditions.^6,7^ However, the Sig-1R receptor is an atypical intracellular chaperone protein localized primarily at the endoplasmic reticulum–mitochondria interface, or mitochondria-associated membrane. It modulates calcium (Ca^2+^) homeostasis, endoplasmic reticulum stress responses, mitochondrial function, and inflammatory signaling, all of which are crucial for neuronal survival and neuroprotection.^10–13^ These distinct but convergent roles of MAO-B and Sig-1R support their potential as complementary targets in neurodegenerative and mood disorders.^4^

Despite the therapeutic value of MAO-B and Sig-1R modulation, discovering ligands that effectively engage both targets remains challenging due to their structural divergence. MAO-B is a mitochondrial enzyme with a well-defined catalytic cavity, whereas Sig-1R is an intracellular chaperone with a deeply buried, highly hydrophobic β-barrel binding pocket.^14,15^ The high-resolution structure of Sig-1R has revealed a homotrimeric architecture with a solvent-occluded binding site that imposes strict physicochemical requirements on ligands. Effective Sig-1R ligands often possess compact, lipophilic scaffolds and a protonatable basic nitrogen that anchors to a conserved Glu172 residue.^4,14,15^ Thus, designing dual-acting molecules, thus, requires careful optimization of lipophilicity, molecular size, and the inclusion of pharmacophoric elements that accommodate both binding sites.

### 3.2 Data Acquisition and Curation

Data for MAO-B and Sig-1R were retrieved from the ChEMBL database and subjected to a rigorous curation process, resulting in final data sets comprising 1873 compounds for MAO-B and 454 compounds for Sig-1R, which were subsequently used for the construction of the classification models.

### 3.3 Descriptor Calculation and Analyses

A total of 5765 descriptors (2D and 3D) were calculated for each data set using AlvaDesc. Descriptors with missing values, low variance, or high correlation were removed during the preprocessing. The MAO-B model was constructed using 10 descriptors, whereas the Sig-1R model employed 13 descriptors. Figure 1 presents the relative importance of each descriptor, estimated by permutation analysis, together with their chemical interpretation based on the training set.

**Figure 1:**
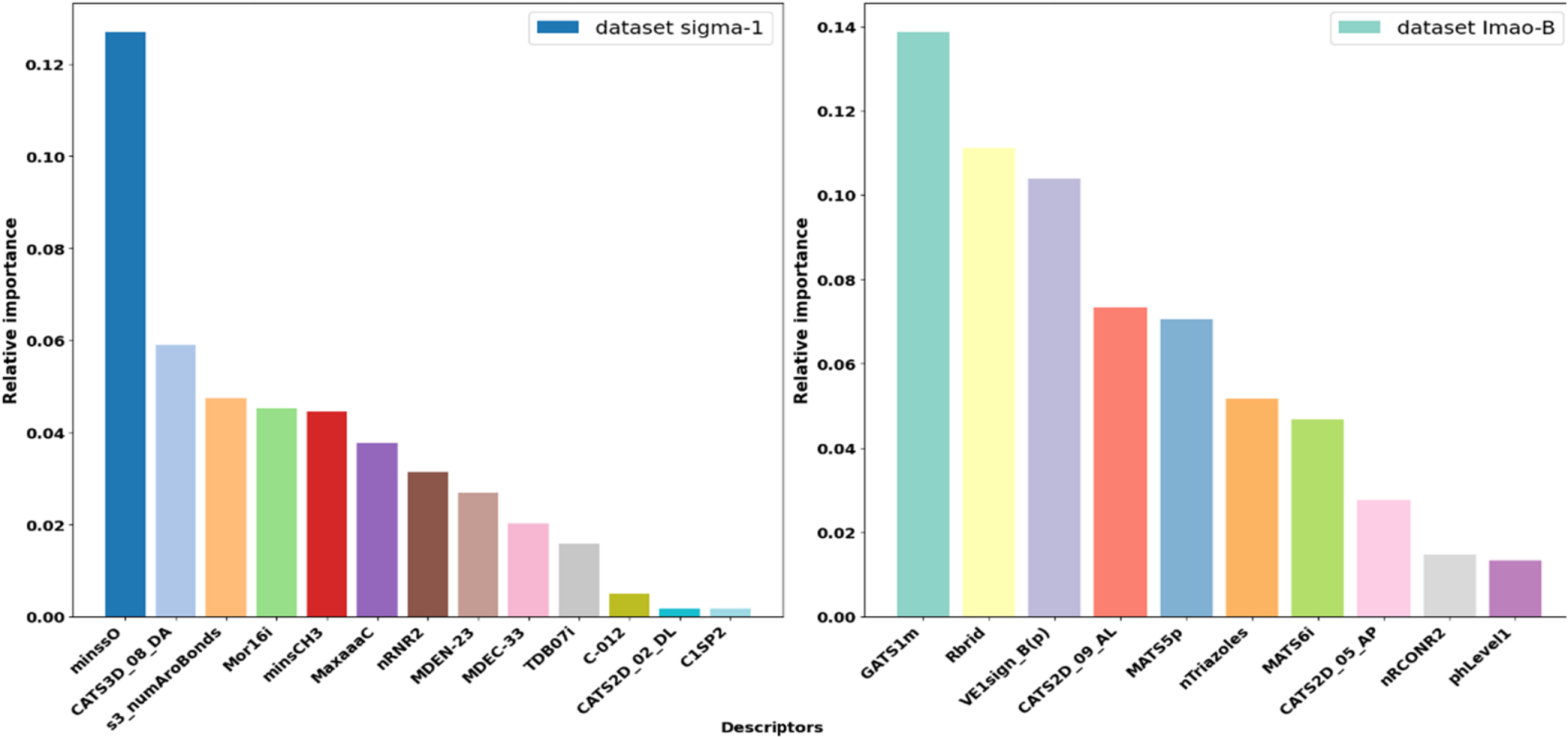
Relative contribution of molecular descriptors to the MAO-B and Sig-1R models developed using the SVM Learner algorithm.

In addition, each descriptor was evaluated based on its mean value within the active and inactive classes of the data sets to infer the direction of descriptor trends. Tables S1 and S2 report the mean values and indicate the tendency of each descriptor for the active and inactive pIC_50_ and p*K_i_* classes, respectively. For the MAO-B model, 10 molecular descriptors were selected, capturing complementary information related to topological mass distribution, structural complexity, and electronic properties (Table S3). In particular, activity was favored by topological mass-distribution patterns (GATS1m, with lower values in the active class), consistent with structures enriched in heteroatoms and higher molecular mass, and by increased polycyclic rigidity/complexity (Rbrid, with higher values in actives), suggesting that more rigid, ring-connected scaffolds enhance biological activity.^41–44^ Descriptors related to polarizability (e.g., VE1sign_B­(p)) did not show isolated statistical significance but contributed jointly to classification, in agreement with the nonlinear nature of the SVM algorithm. Additional descriptors highlighted the relevance of the spatial distribution of hydrogen-bond donors and acceptors, specific topological distances (5–9 bonds),^45^ the presence of tertiary amides (R–CO–NR_2_), favorable electronic features, and the absence of triazole rings.

For the Sig-1R model, 13 descriptors were selected, primarily reflecting polarity, ionization potential, and 3D spatial arrangements (Table S3). Activity was mainly associated with higher contributions of minssO, which captures the minimum electronic state of oxygen in ether (−O−) fragments,^46,47^ emphasizing the role of molecular polarity, and with donor–acceptor spatial patterns at 8–9 Å (CATS3D_08_DA).^48^ Descriptors related to aromaticity (s3_numAroBonds) were also relevant, although without a clear class-specific trend. For Sig-1R, activity was disfavored by the presence of CR2 × 2 fragments, which correspond to carbon atoms simultaneously bonded to two heteroatoms (such as O, N, or S). That is typically associated with highly polarized or structurally rigid chemical environments, as well as by the presence of methyl groups (−CH_3_), donor–lipophilic atom pairs at distances of 8–9 Å, secondary and tertiary amines, and excessive aromaticity, characterized by sp^2^ carbons bonded exclusively to other carbons.^48–50^

### 3.4 Model Development Performance

After data processing and curation, the final training sets comprised 102 compounds for MAO-B and 86 compounds for Sig-1R, while the external test sets contained 26 and 22 compounds, respectively. The performance metrics (Table 1) of the models were obtained for the training, cross-validation, and external test data sets.

**Table 1:** Performance Metrics of the SVM Learner Models in the Training, Validation, and External Test Sets for the MAO-B and Sig-1R Data Sets

data set	model (SVM)	kappa coefficient	precision (%)	sensitivity (%)	specificity (%)	F1 score (%)	accuracy (%)	ROC	MCC
training	MAO-B	0.863	97.83	88.24	98.04	92.78	93.14	0.959	0.867
	Sig-1R	0.860	91.11	95.35	90.70	93.18	93.02	0.944	0.861
cross-validation	MAO-B	0.784	93.48	84.31	94.12	88.66	89.22	0.912	0.788
	Sig-1R	0.837	90.91	93.00	90.70	92.00	91.90	0.936	0.837
external test	MAO-B	0.769	91.70	84.60	92.30	88.00	88.46	0.840	0.771
	Sig-1R	0.818	90.91	90.91	90.91	90.91	90.90	0.975	0.818

In the training set, both models exhibited high performance, with Kappa >0.86, precision >0.91, MCC > 0.86, ROC > 0.94, and accuracy ≈0.93, indicating substantial agreement beyond chance and a strong ability to discriminate between active and inactive compounds for MAO-B and Sig-1R. This performance was maintained in cross-validation and external test sets, with Kappa ≥ 0.77 and accuracy ≥ 0.88, supporting the robustness and generalization capability of the models. The ROC curve plots are presented in Figures S1 and S2.

Exploratory regression models were also evaluated using the descriptors retained in the classification workflow; however, their predictive performance was not considered sufficiently robust for continuous QSAR modeling under the present conditions (Table S4). This finding further supported the use of a classification strategy as the most appropriate approach for compound prioritization in this study.

### 3.5 Virtual Screening

The results of the APD assessment for the MAO-B and Sig-1R virtual screening data sets are summarized in Table S5. Both QSAR models exhibited broad chemical space coverage, with 91.2% of the screened structures falling within the APD for MAO-B and 93.2% for Sig-1R. These results indicate that the developed QSAR models cover approximately 90% of the chemical space represented in the virtual screening library, supporting their robustness and predictive applicability. Based on this high coverage and the previously obtained performance metrics, virtual screening was performed exclusively on structures located within the APD. The outcomes of the QSAR-based virtual screening are reported in Table S6, which summarizes the number of compounds classified as active or inactive for each molecular target. Compounds with predicted dual-target potential were identified as those classified as active by both the MAO-B and Sig-1R models. In total, 11 compounds met this criterion and are listed in Table S7 and Figure 2.

**Figure 2:**
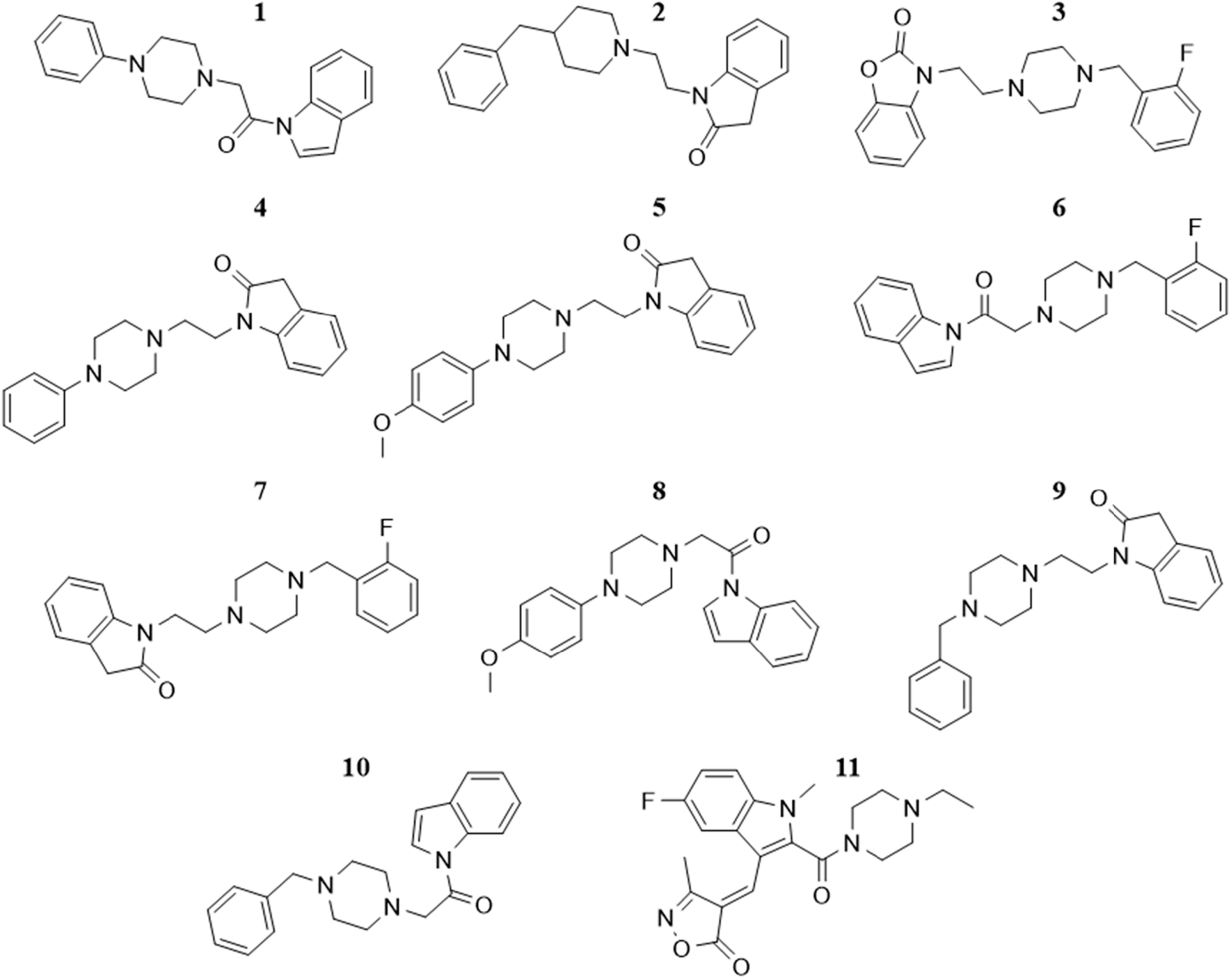
Compounds predicted as potential candidates by both MAO-B and Sig-1R QSAR models.

### 3.6 Molecular Docking

The crystallographic structures were selected based on structural quality, presence of pharmacologically relevant cocrystallized ligands, and suitability for molecular recognition studies. For the Sig-1R receptor, the human structure PDB ID: 5HK1, cocrystallized with the selective antagonist PD144418 and solved at 2.51 Å resolution, was chosen. This structure reveals the trimeric organization of Sig-1R and a deep, conformationally flexible ligand-binding cavity, providing a biologically relevant model for Sig-1R ligand recognition.^15^ For MAO-B, the human structure PDB ID: 2V5Z, cocrystallized with the selective and reversible inhibitor safinamide and solved at 1.60 Å resolution, was employed. This structure accurately depicts the bipartite active site (entrance and substrate cavities) and the orientation of the FAD cofactor, offering a robust framework for docking validation and comparative studies of MAO-B inhibitors.^51^

### 3.7 Molecular Docking Analysis of the Curated Library

Molecular docking analysis was performed on a curated library of 147 chemical structures, each annotated with a structural index and InChIKey and preprocessed as described in the Materials and Methods section, including energy minimization, protonation state assignment at pH 7.4, and PDBQT file generation. This same curated library was also evaluated using QSAR models. Thus, docking was used as a complementary structure-based approach to comparatively rank compounds within each target and to assess the plausibility of their predicted binding modes, rather than as direct evidence of binding or absolute binding affinity. Molecular docking was carried out using AutoDock Vina, generating up to 10 poses per compound, ranked by predicted binding affinity and analyzed through visual inspection of key interactions with the active sites of MAO-B and the Sig-1R receptor. Docking parameters followed the protocol validated by redocking, with rigid receptors and fully flexible ligands, using grid centers at *X* = 11.755, *Y* = 37.191, *Z* = – 34.868 for Sig-1R and *X* = 51.886, *Y* = 156.453, *Z* = 28.559 for MAO-B, a grid size of 25 × 25 × 25 Å^3^, and a high exhaustiveness value of 128 to enhance conformational sampling. Redocking of the Sig-1R reference ligand PD144418 (PDB ID: 5HK1) yielded a lowest-energy pose with a predicted binding affinity of −9.9 kcal·mol^–1^ and an RMSD of 1.478 Å relative to the crystallographic conformation, supporting the suitability of the docking protocol for comparative pose analysis in reproducing the characteristic binding mode of Sig-1R ligands (Figure S3 and Table S8).

Redocking of the reference ligands validated the docking protocols for both targets, yielding a predicted binding affinity of −9.9 kcal·mol^–1^ for PD144418 at the Sig-1R receptor with an RMSD of 1.478 Å, and −10.14 kcal·mol^–1^ for safinamide at MAO-B with an RMSD of 0.509 Å. Thus, confirming high reproducibility of the crystallographic binding modes (Tables S8 and S9 and Figures S3 and S7). To further strengthen the structural validation of the docking setup, additional redocking and cross-docking experiments were performed using crystallographic ligands from independent Sig-1R and MAO-B complexes. The protocol reproduced the crystallographic binding modes and accommodated the additional ligands within the receptor structures used in the main study, providing further structural support for the comparative docking analysis (Supporting Information, Tables S13–S16 and Figures S21–S24). Virtual screening results revealed that the top 10 Sig-1R ligands displayed more favorable predicted affinities (−11.1 to −12.4 kcal·mol^–1^) than the crystallographic reference (Table S10), suggesting favorable docking scores and structurally plausible binding modes within the adopted docking framework, while MAO-B screening identified compounds with affinities ranging from −11.6 to −10.2 kcal·mol^–1^, comparable to or exceeding safinamide (Table S11).

Structural analysis showed that Sig-1R ligands consistently occupied the same binding region as PD144418, establishing conserved hydrogen-bond interactions with Glu172 and Asp126, complemented by hydrophobic and π–π interactions with residues such as Val84, Met93, Leu95, Leu105, Ile124, Leu182, Trp89, Phe107, and Trp164, reinforcing protocol reliability (Figures S4 and S5). In MAO-B, prioritized ligands were positioned in front of the FAD redox center (N5–C4a region), sterically blocking substrate access in agreement with a competitive and reversible inhibition mechanism, and were stabilized by hydrophobic and aromatic interactions within the bipartite active site (Figures S6 and S8). Comparative analysis across both targets identified recurrent ligands among the prioritized candidates (compounds SLIKVHAAPXFGRD-UHFFFAOYSA-N, MQIBQAJHZNOHAA-UHFFFAOYSA-N, SWXAVUNIIFCCSX-JLUADYMRSA-N, and SXGFSUBZLBBWMV-YDZHTSKRSA-N), which exhibited consistently favorable predicted docking scores for Sig-1R (−11 kcal·mol^–1^) and MAO-B (−11.3 to −10.2 kcal·mol^–1^), suggesting structural convergence in molecular recognition despite distinct binding-site architectures (Figure 3). These ligands share lipophilic aromatic cores combined with protonatable or polar functionalities that enable electrostatic anchoring at Sig-1R (notably via Glu172) and modulatory polar interactions at MAO-B (often involving Gln206 and structured water molecules), supporting the rationale for a multitarget virtual screening strategy.

**Figure 3:**
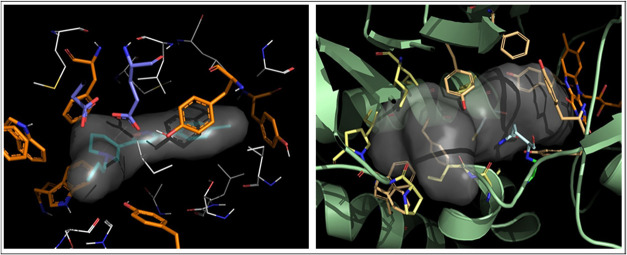
Comparison of the binding cavities of the Sig-1R receptor (left) and monoamine oxidase B (MAO-B) (right). The gray molecular surfaces illustrate similar cavity size and volume and emphasize the hydrophobic, aromatic-rich character of both binding sites. This shared structural and physicochemical profile supports the recognition of ligands with common chemical features and provides a rationale for identifying compounds with dual binding potential across the two targets.

### 3.8 Molecular Dynamics Simulation

Based on the docking results, compounds 102 (SLIKVHAAPXFGRD-UHFFFAOYSA-N) and 072 (MQIBQAJHZNOHAA-UHFFFAOYSA-N) were selected for molecular dynamics simulations with MAO-B and Sig-1R to evaluate the short-term structural stability of the protein–ligand complexes over 150 ns; this time scale was employed as a structural filter to verify the maintenance of docking-predicted binding modes under dynamic conditions. Stability was assessed using RMSD (root-mean-square deviation, to monitor structural deviation), RMSF (root-mean-square fluctuation, to indicate residue flexibility), radius of gyration (Rg), monitored to assess the global structural integrity and system equilibration during the simulation, and the number of hydrogen bonds. Protein RMSD profiles showed an initial increase followed by a stable plateau with minor fluctuations, indicating initial accommodation and subsequent structural stabilization, with convergence observed after approximately 75 ns (Figures S9 and S10). For clarity, the RMSD values in Figure S9 are reported in Å; thus, values above 6 Å correspond to approximately 0.6 nm and should not be interpreted as 0.6 Å.

Ligand RMSD values remained below 2.5 Å, suggesting the retention of the binding mode despite expected conformational adjustments (Figures S11 and S12). RMSF analysis revealed higher flexibility limited to the *N*- and *C*-termini (amino and carboxyl ends of the protein), while residues in the binding site displayed low fluctuations, supporting local complex stability (Figures S13 and S14). Consistently, *R*_g_ values were monitored only as a general descriptor of structural compactness during the simulated time scale and were not interpreted as evidence of long-term conformational stability (Figures S15 and S16). The H bond analysis highlighted target-dependent behavior: in MAO-B, the reference ligand maintained H bonds throughout the simulation, indicating strong anchoring, whereas in Sig-1R, fewer hydrogen bonds were observed, suggesting a predominant role of hydrophobic and aromatic interactions (Figures S17 and S18). Analysis of representative MD poses showed that, in MAO-B, residues such as Cys172 and Ile316 engage in recurrent hydrophobic interactions with compounds 102 and 072 (Figures 4 and S19), while in Sig-1R, interactions with Tyr103, Leu105, and Leu182 were predominant, also mainly hydrophobic and aromatic, in agreement with literature reports (Figure S20). Overall, these results suggest that compounds 102 and 072 (Table S12) can maintain stable binding modes over the simulated time scale, supporting their prioritization as potential dual-target candidates for future experimental validation.

**Figure 4:**
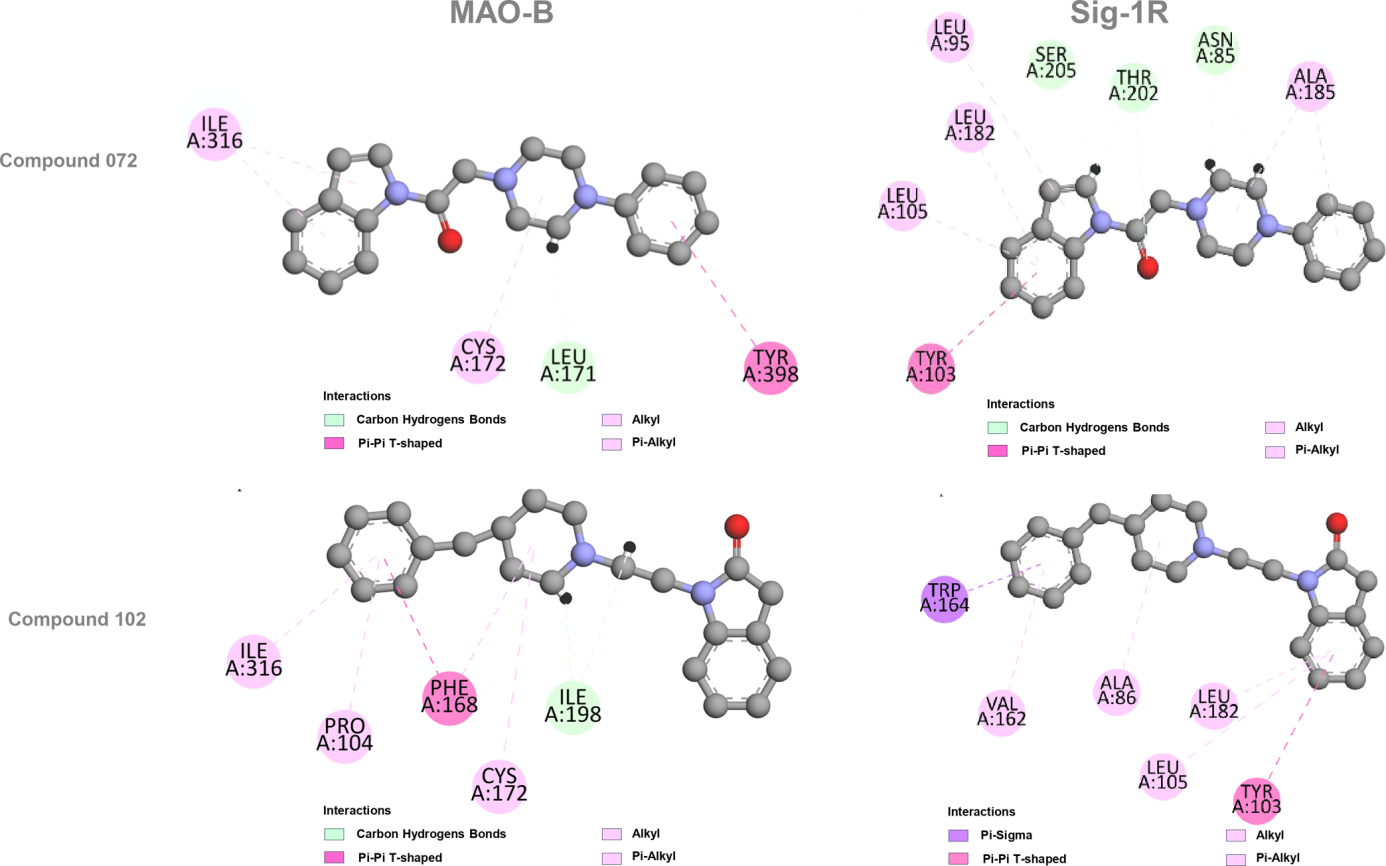
2D interaction maps of the most representative protein–ligand complexes obtained from molecular dynamics simulations for MAO-B (left) and Sig-1R (right) targets.

### 3.9 Structure–Activity Relationship of Active Compounds

The integrated analysis of QSAR, virtual screening, molecular docking, expanded docking validation, and molecular dynamics reveals a clear structural convergence among compounds prioritized for MAO-B and Sig-1R modulation (Tables S1–S3, S9–S11, and S13–S16 and Figures S4–S24). Overall, the most promising ligands share a modular pharmacophore composed of a protonatable basic center (piperidine or piperazine), lipophilic aromatic moieties, and a short spacer controlling their relative orientation, a motif widely reported for both MAO-B and Sig-1R receptor ligands.^52,53^ For Sig-1R (Figures S4, S5, and S20–S22), this architecture favors electrostatic anchoring via conserved acidic residues such as Glu172 and Asp126, together with hydrophobic and aromatic contacts within a predominantly apolar cavity.^13,15,54^ Conversely, in MAO-B (Figures S6–S8, S19, S23, and S24), the additional presence of carbonyl groups (amide or lactam) conjugated to aromatic systems promotes optimal accommodation within the bipartite active site and stabilizing interactions near the FAD cofactor, in agreement with crystallographic and structure–activity relationship (SAR) studies of reversible MAO-B inhibitors such as safinamide and indole-based scaffolds.^51,55,56^ Compounds 072 and 102, prioritized by QSAR and docking analyses, maintained stable predicted binding modes over 150 ns of molecular dynamics, as indicated by convergent RMSD, RMSF, radius of gyration, monitored as a general descriptor of structural compactness and hydrogen-bond profiles (Figures S9–S18), establishing recurrent interactions with Cys172 and Ile316 in MAO-B and Tyr103, Leu105, and Leu182 in Sig-1R. Collectively, these findings indicate that dual activity is favored by the combination of a distal cationic center, lipophilic aromatic modules, and strategically positioned polar functionalities, enabling conformational adaptation to both the compact Sig-1R cavity and the elongated catalytic channel of MAO-B, and supporting previous multitarget design strategies reported in the literature.^57,58^

## 4. Conclusions

This study highlights indole- and oxindole-based piperazine scaffolds as promising chemical architectures for the rational exploration of dual modulation of MAO-B and Sig-1R in the context of multitarget neuroprotective drug discovery. Through the integration of descriptor-driven machine learning models with structure-based simulations, we demonstrate that these molecular frameworks combine aromatic planarity, electronic complementarity, and conformational adaptability that are compatible with the molecular recognition requirements of both targets. The descriptor-driven analysis identified that the precise orientation of the *N*-aryl piperazine motif relative to the indole/oxindole system is a primary determinant of dual bioactivity. Thus, rather than emphasizing individual binding events, this work establishes general SAR principles, showing that the spatial organization between aromatic cores and distal basic centers is a key determinant of multitarget compatibility. The convergence observed among QSAR predictions, docking poses, and molecular dynamics results supports the relevance of this scaffold class as a promising starting point for multitarget design.

Although exploratory regression analyses were assessed, their predictive performance remained limited in the present data set, indicating that future regression-based extensions will likely require more homogeneous activity data and descriptor selection specifically optimized for continuous modeling. Together, these findings reinforce the value of integrated computational strategies to guide future synthesis and experimental evaluation of multitarget ligands for complex central nervous system disorders.

## Supplementary Material



## Data Availability

All supplementary data supporting the findings of this study are publicly available on Zenodo. The deposited materials include all compounds classified as active against MAO-B and Sig-1R, provided in.mol file format, detailed tables for the training and test data sets, and the complete KNIME workflows used for model development, classification, and prediction. In addition, the data set of compounds employed in the virtual screening campaign is also provided. The data can be accessed at: DOI: https://zenodo.org/uploads/18500367.

## Notes

Trend of descriptor averages in relation to active and inactive classes for the MAO-B training set (Table S1); trend of descriptor averages in relation to active and inactive classes for the Sig-1R training set (Table S2); descriptor descriptions for the MAO-B and Sig-1R models (Table S3); exploratory regression analyses performed using descriptors selected from the classification workflow (Table S4); applicability domain results for the virtual screening libraries per model (Table S5); screening results and number of structures classified as active by each model (Table S6); structures classified as active by both MAO-B and Sig-1R models (Table S7); redocking results of the reference ligand within the Sig-1R receptor using AutoDock Vina (Table S8); redocking results of the reference ligand within the MAO-B active site using AutoDock Vina (Table S9); virtual screening results for the top 10 ranked ligands targeting the Sigma-1 receptor (Sig-1R) using AutoDock Vina (Table S10); virtual screening results for the ten top-ranked ligands targeting MAO-B using AutoDock Vina (Table S11); summary of compounds 102 and 072 showing structurally stable complexes over the simulated time scale and supporting their prioritization as potential dual-activity candidates against MAO-B and Sig-1R (Table S12); AutoDock Vina results for redocking of the 5HK2 crystallographic ligand into its native Sig-1R receptor (Table S13); AutoDock Vina results for cross-docking of the 5HK2 crystallographic ligand into the 5HK1 Sig-1R receptor (Table S14); AutoDock Vina results for redocking of the 2 V61 crystallographic ligand into its native MAO-B receptor (Table S15); AutoDock Vina results for cross-docking of the 2 V61 crystallographic ligand into the 2 V5Z MAO-B receptor (Table S16); receiver operating characteristic (ROC) curve for the Sig-1R model, including the area under the curve (AUC) for the training, test, and cross-validation data sets (Figure S1); receiver operating characteristic (ROC) curve for the MAO-B model, including the area under the curve (AUC) for the training, test, and cross-validation data sets (Figure S2); redocking result of the reference ligand PD144418 within the active site of the Sigma-1 receptor (Sig-1R) (Figure S3); superposition of the docking poses of the ten top-ranked ligands selected by virtual screening within the active site of the Sig-1R receptor (Figure S4); active site of the Sig-1R receptor highlighting the main residues involved in ligand recognition (Figure S5); representation of the binding mode of safinamide within the active site of monoamine oxidase B (MAO-B) (Figure S6); redocking result of the reference ligand safinamide within the active site of monoamine oxidase B (MAO-B) (Figure S7); superposition of the ten best docking compounds retrieved from virtual screening within the active site of monoamine oxidase B (MAO-B) (Figure S8); RMSD of the MAO-B protein complexed with different ligands during 150 ns (Figure S9); RMSD of the Sig-1R protein complexed with different ligands during 150 ns (Figure S10); ligand RMSD during 150 ns of simulation within the MAO-B protein (Figure S11); ligand RMSD during 150 ns of simulation within the Sig-1R protein (Figure S12); RMSF of the MAO-B protein complexed with different ligands during 150 ns (Figure S13); RMSF of the Sig-1R protein complexed with different ligands during 150 ns (Figure S14); radius of gyration of the MAO-B protein complexed with different ligands during 150 ns (Figure S15); radius of gyration of the Sig-1R protein complexed with different ligands during 150 ns (Figure S16); number of hydrogen bonds in the MAO-B protein with different ligands during 150 ns (Figure S17); number of hydrogen bonds in the Sig-1R protein with different ligands during 150 ns (Figure S18); interactions for the most representative complexes during the molecular dynamics simulation for the MAO-B target (Figure S19); interactions for the most representative complexes during the molecular dynamics simulation for the Sig-1R target (Figure S20); superposition of the crystallographic pose of the 5HK2 ligand and the best redocked pose in the Sig-1R binding site (Figure S21); cross-docking of the crystallographic ligand from 5HK2 into the 5HK1 Sig-1R receptor (Figure S22); superposition of the crystallographic pose of the 2 V61 ligand and the best redocked pose in the MAO-B active site (Figure S23); and cross-docking of the crystallographic ligand from 2 V61 into the 2 V5Z MAO-B receptor (Figure S24) (PDF)

## Abbreviations

APD: applicability domain
Å: angstrom
**Ca** ^**2+**^: calcium ion
Cα: α carbon
CNS: central nervous system
ER: endoplasmic reticulum
FAD: flavin adenine dinucleotide
IC_50_: half maximal inhibitory concentration
K*_i_*: inhibition constant
κ: Cohen’s kappa coefficient
LASSO: least absolute shrinkage and selection operator
ROC: receiver operating characteristic
MCC: Matthews correlation coefficient
MAO-B: monoamine oxidase B
MD: molecular dynamics
