# Supplementary material for: Machine Learning-Driven Discovery of Indole/Oxoindole–Piperazine Scaffolds as Dual MAO-B/Sig-1R Ligands for Neurodegenerative Disorders
Source: J Chem Inf Model. 2026 Jul 7;66(14):7889–99. doi: 10.1021/acs.jcim.6c00379 (PMC13417874; doi:10.1021/acs.jcim.6c00379)
Supplement: Supplementary file 1 [file ci6c00379_si_001.pdf]

# Supporting Information

# Machine Learning-Driven Discovery of Indole/Oxoindole–Piperazine Scaffolds as Dual MAO-B/Sig-1R Ligands for Neurodegenerative Disorders

*Ulisses Candido da Silva[a], Miller Santos Ferreira[a], Eduardo Borba Alves[b], Valéria Vieira Moura Paixão[b], Igor Rodrigues Lapa[a], Micaela M. Barbosa Nogueira[a], Ricardo Pereira Rodrigues[e], Wanda Pereira Almeida[e], Marisi Gomes Soares[a], Daniela A. Chagas-Paula[c], Albert Katchborian-Neto[d], Tiago Branquinho Oliveira[b] and Danielle Ferreira Dias[a*]*

[a]Institute of Chemistry, Federal University of Alfenas (UNIFAL), Alfenas, Minas Gerais, 37130-00, Brazil.

[b]Department of Pharmacy, Federal University of Sergipe (UFS), São Cristóvão, Sergipe, 49107-230, Brazil.

[c]Department of Chemistry, Federal University of Juiz de Fora (UFJF), Juiz de Fora, Minas Gerais, 36036-900, Brazil.

[d]Department of Chemistry, Federal University of São Carlos (UFSCAR), São Carlos, São Paulo, 13565-905, Brazil.

[e]School of Pharmaceutical Sciences, State University of Campinas (UNICAMP), Campinas, São Paulo, 13083-871, Brazil

*Corresponding author at Institute of Chemistry, Federal University of Alfenas, Gabriel Monteiro da Silva 700, 37130-001 Alfenas, Minas Gerais, Brazil.

E-mail address: danielle.dias@unifal-mg.edu.br

## TABLE OF CONTENTS

**Table S1.** Trend of descriptor averages in relation to active and inactive classes for the MAO-B training set.

**Table S2.** Trend of descriptor averages in relation to active and inactive classes for the Sig-1R training set.

**Table S3**. Descriptor descriptions for the MAO-B and Sig-1R models.

**Table S4.** Exploratory regression analyses performed using descriptors selected from the classification workflow.

**Table S5.** APD (Applicability Domain) results for the virtual screening libraries per model.

**Table S6.** Screening results and number of structures classified as active by each model.

**Table S7**. Structures classified as active by both MAO-B and Sig-1R models.

**Table S8.** Redocking results of the reference ligand within the Sig-1R receptor using AutoDock Vina. Predicted binding affinity values (kcal·mol$^{-1}$) were used as an additional criterion) are reported for each binding mode, along with the corresponding structural deviations (RMSD l.b. and RMSD u.b.), which represent the minimum and maximum geometric distances of each pose relative to the lowest-energy docking pose, as defined by AutoDock Vina

**Table S9**. Redocking results of the reference ligand within the MAO-B active site using AutoDock Vina. Predicted binding affinity values (kcal·mol$^{-1}$) were used as an additional criterion) are reported for each binding mode, along with the corresponding structural deviations (RMSD l.b. and RMSD u.b.), which represent the minimum and maximum geometric distances of each pose relative to the lowest-energy docking pose, as defined by AutoDock Vina.

**Table S10**. Virtual screening results for the top 10 ranked ligands targeting the Sigma-1 receptor (Sig-1R) using AutoDock Vina.

**Table S11**. Virtual screening results for the ten top-ranked ligands targeting MAO-B using AutoDock Vina.

**Table S12**. Compounds 102 and 072 showed structurally stable complexes over the simulated timescale, supporting their prioritization as dual-activity potential candidates against MAO-B and Sig-1R.

**Table S13.** AutoDock Vina results for redocking of the 5HK2 crystallographic ligand into its native Sig-1R receptor.

**Table S14.** AutoDock Vina results for cross-docking of the 5HK2 crystallographic ligand into the 5HK1 Sig-1R receptor.

**Table S15.** AutoDock Vina results for redocking of the 2V61 crystallographic ligand into its native MAO-B receptor.

**Table S16.** AutoDock Vina results for cross-docking of the 2V61 crystallographic ligand into the 2V5Z MAO-B receptor.

**Figure S1.** Receiver Operating Characteristic (ROC) curve for the Sig-1R model. The graph shows the area under the curve (AUC), which corresponds to the model's performance on the training, test, and cross-validation datasets.

**Figure S2.** Receiver Operating Characteristic (ROC) curve for the MAO-B model. The graph shows the area under the curve (AUC), which corresponds to the model's performance on the training, test, and cross-validation datasets.

**Figure S3**. Redocking result of the reference ligand PD144418 within the active site of the Sigma-1 receptor (Sig-1R). The lowest-energy pose predicted by AutoDock Vina is shown in yellow, while the crystallographic reference ligand is displayed for structural comparison, yielding an RMSD of 1.478 Å.

**Figure S4.** Superposition of the docking poses of the ten top-ranked ligands selected by virtual screening within the active site of the Sig-1R receptor.

**Figure S5.** Active site of the Sig-1R receptor highlighting the main residues involved in ligand recognition.

**Figure S6**. Representation of the binding mode of safinamide within the active site of monoamine oxidase B (MAO-B). The ligand (shown in green) is fitted within the catalytic channel, occupying the hydrophobic cavity adjacent to the FAD cofactor (shown in orange, right) and interacting with the MAO-B selectivity loop (shown in orange, left), which contributes to the differentiation between the MAO-A and MAO-B isoforms and is associated with the selective recognition of inhibitors.

**Figure S7.** Redocking result of the reference ligand safinamide within the active site of monoamine oxidase B (MAO-B). The lowest-energy pose predicted by AutoDock Vina is shown in yellow, while the crystallographic reference ligand is displayed for structural comparison. Structural alignment of the two conformations yielded an RMSD value of 0.509 Å, indicating accurate reproduction of the experimental binding mode.

**Figure S8.** Superposition of the ten best docking compounds retrieved from virtual screening within the active site of monoamine oxidase B (MAO-B). The different ligand conformations (represented in distinct colours) highlight the preferential occupation of the hydrophobic catalytic channel, showing good steric complementarity with the active site cavity (surface shown in grey). The FAD cofactor (shown in orange) is displayed for spatial reference, emphasising the proximity of the poses to the catalytic region of the enzyme.

**Figure S9**. RMSD of the MAO-B protein complexed with different ligands during 150 ns.

**Figure S10.** RMSD of the Sig-1R protein complexed with different ligands during 150 ns.

**Figure S11.** Ligand RMSD during 150 ns of simulation within the MAO-B protein.

**Figure S12**. Ligand RMSD during 150 ns of simulation within the Sig-1R protein.

**Figure S13.** RMSF of the MAO-B protein complexed with different ligands during 150 ns.

**Figure S14.** RMSF of the Sig-1R protein complexed with different ligands during 150 ns.

**Figure S15**. Radius of Gyration ($R_g$) of the MAO-B protein complexed with different ligands during 150 ns.

**Figure S16.** Radius of Gyration ($R_g$) of the Sig-1R protein complexed with different ligands during 150 ns.

**Figure S17.** Number of H-bonds in the MAO-B protein with different ligands during 150 ns.

**Figure S18**. Number of H-bonds in the Sig-1R protein with different ligands during 150 ns.

**Figure S19.** Interactions for the most representative complexes during the molecular dynamics simulation for the MAO-B target.

**Figure S20.** Interactions for the most representative complexes during the molecular dynamics simulation for the Sig-1R target.

**Figure S21.** Superposition of the crystallographic pose of the 5HK2 ligand and the best redocked pose.

**Figure S22.** Cross-docking of the crystallographic ligand from 5HK2 into 5HK1.

**Figure S23.** Superposition of the crystallographic pose of the 2V61 ligand and the best redocked pose.

**Figure S24.** Cross-docking of the crystallographic ligand from 2V61 into 2V5Z.

**Table S1**.Trend of descriptor averages in relation to active and inactive classes for the MAO-B training set.

| Descriptor | Statistical test | *p*-value | Class 1 | Class 0 | Class $pIC_{50}$ trend |
|---|---|---|---|---|---|
| Rbrid | Wilcoxon | 6.09E-6 | 1.0* | 0.0* | Favors Class 1 |
| VE1sign_B(p) | Wilcoxon | 0.051 | 0.14* | 0.06* | Not significant |
| MATS5p | Wilcoxon | 0.00154 | 0.07* | 0.01* | Favors Class 1 |
| MATS6i | T-Test | 0.06 | 0.03** | -0.01** | Not significant |
| GATS1m | T-Test | 6.22E-7 | 0.69** | 0.80** | Favors Class 0 |
| nRCONR2 | Wilcoxon | 0.16 | 0.0* | 0.0* | Not significant |
| nTriazoles | Wilcoxon | 4.87E-4 | 0.0* | 0.0* | Equal |
| CATS2D_05_AP | Wilcoxon | 0.16 | 0.0* | 0.0* | Not significant |
| CATS2D_09_AL | Wilcoxon | 7.67E-5 | 4.0* | 1.0* | Favors Class 1 |
| phLevel1 | Wilcoxon | 0.0065 | 0.0* | 0.0* | Equal |

*** median ** mean**

**Table S2**. Trend of descriptor averages in relation to active and inactive classes for the Sig-1R training set.

| Descriptors | Statistical test | *p*-value | Class 1 | Class 0 | Trend Class pKi |
|---|---|---|---|---|---|
| C1SP2 | Wilcoxon | 6.70E-5 | 0.0[a] | 1.0[a] | Favors Inactive (0) |
| TDB07i | T-Test | 4.59E-5 | 7.99[b] | 8.34[b] | Favors Inactive (0) |
| Mor16i | T-Test | 0.01 | 1.14[b] | 0.75[b] | Favors Active (1) |
| nRNR2 | Wilcoxon | 5.96E-4 | 1.0[a] | 1.0[a] | Equal[c] |
| C-012 | Wilcoxon | 0.65 | 0.0[a] | 0.0[a] | Not Significant |
| minsCH3 | Wilcoxon | 0.15 | 1.60[a] | 1.80[a] | Not Significant [d] |
| minssO | Wilcoxon | 0.0039 | 5.28[a] | 0.0[a] | Favors Active (1) |
| MaxaaaC | Wilcoxon | 0.017 | 0.0[a] | 0.0[a] | Equal |
| CATS2D_02_DL | Wilcoxon | 0.0048 | 0.0[a] | 1.0[a] | Favors Inactive (0) |
| CATS3D_08_DA | Wilcoxon | 0.0039 | 0.0[a] | 0.0[a] | Equal |
| MDEC-33 | Wilcoxon | 3.11E-4 | 3.15[a] | 6.77[a] | Favors Inactive (0) |
| MDEN-23 | Wilcoxon | 7.99E-4 | 0.0[a] | 0.23[a] | Favors Inactive (0) |
| s3_numAroBonds | Wilcoxon | 0.021 | 0.0[a] | 0.0[a] | Equal |

[a] median [b]mean; [c]No median difference [d]significant median difference

**Table S3**. Descriptor descriptions for the MAO-B and Sig-1R models.

| Descriptor - MAO-B | Description |
|---|---|
| CATS2D_09_AL | CATS2D Acceptor-Lipophilic at lag 09 |
| Rbrid | ring bridge count |
| GATS1m | Geary autocorrelation of lag 1 weighted by mass |
| VE1sign_B(p) | coefficient sum of the last eigenvector from Burden matrix weighted by polarizability |
| CATS2D_05_AP | CATS2D Acceptor-Positive at lag 05 |
| MATS5p | Moran autocorrelation of lag 5 weighted by polarizability |
| phLevel1 | number of neighbouring N/O atoms of the chiral centre (level 1) |

| | |
|---|---|
| nRCONR2 | number of tertiary amides (aliphatic) |
| MATS6i | Moran autocorrelation of lag 6 weighted by ionization potential |
| nTriazoles | number of Triazoles |
| **Descriptor – Sig-1R** | **Description** |
| TDB07i | 3D Topological distance based descriptors – lag 7 weighted by ionization potential |
| MDEC-33 | molecular distance edge between all tertiary carbons |
| minssO | Minimum ssO [-O-] |
| s3_numAroBonds | number of aromatic bonds of the substituent 3 |
| Mor16i | signal 16 / weighted by ionization potential |
| minsCH3 | Minimum sCH3 |
| nRNR2 | number of tertiary amines (aliphatic) |
| CATS2D_02_DL | CATS2D Donor-Lipophilic at lag 02 |
| C1SP2 | Sp2 carbon bonded to one carbon atom |
| MDEN-23 | molecular distance edge between all secondary and tertiary nitrogens |
| MaxaaaC | Maximum aaaC |
| C-012 | CR2X2 |
| CATS3D_08_DA | CATS3D Donor-Acceptor BIN 08 (8.000 – 9.000 Å) |

**Table S4.** Exploratory regression analyses performed using descriptors selected from the classification workflow.

| *Models* | *Metrics* | *Values* | *RMSE* | *MAE* |
|---|---|---|---|---|
| SMOreg (MAOB) | R2 | 0.568 | 1.329 | 0.993 |
| | P2 | -0.130 | 2.694 | 1.933 |
| | Q2 | 0.465 | 1.479 | 1.149 |
| M5P (MAOB) | R2 | 0.528 | 1.389 | 1.137 |
| | P2 | -0.013 | 2.552 | 1.988 |
| | Q2 | 0.436 | 1.518 | 1.207 |
| ANN (MAOB) | R2 | 0.600 | 1.278 | 1.080 |
| | P2 | 0.290 | 2.137 | 1.648 |
| | Q2 | 0.071 | 1.948 | 1.412 |
| LinearRegression (MAOB) | R2 | 0.598 | 1.281 | 1.060 |
| | P2 | 0.015 | 2.516 | 1.896 |
| | Q2 | 0.427 | 1.530 | 1.249 |
| SMOreg (Sig-1R) | R2 | 0.523 | 0.993 | 0.643 |
| | P2 | 0.524 | 0.770 | 0.640 |
| | Q2 | 0.469 | 1.046 | 0.728 |
| M5P (Sig-1R) | R2 | 0.650 | 0.850 | 0.614 |
| | P2 | 0.481 | 0.804 | 0.677 |
| | Q2 | 0.428 | 1.086 | 0.793 |
| ANN (Sig-1R) | R2 | 0.605 | 0.902 | 0.712 |
| | P2 | -4.223 | 2.550 | 2.136 |
| | Q2 | -1.466 | 2.256 | 1.714 |

| | | | | |
|---|---|---|---|---|
| LinearRegression (Sig-1R) | R2 | 0.526 | 0.989 | 0.725 |
| | P2 | 0.594 | 0.711 | 0.580 |
| | Q2 | 0.355 | 1.154 | 0.841 |

**Table S5**. APD (Applicability Domain) results for the virtual screening libraries per model.

| Models | Database | APD structures | Percentage (%) |
|---|---|---|---|
| MAO-B | 147 | 134 | 91.2% |
| Sig-1R | 147 | 137 | 93.2% |

**Table S6:** Screening results and number of structures classified as active by each model.

| Target | Active structures | Inactive structures |
|---|---|---|
| MAO-B | 114 | 33 |
| Sig-1R | 14 | 133 |

**Table S7**: Structures classified as active by both MAO-B and Sig-1R models.

| Index | Compound Index | InChIKey | Classified structures |
|---|---|---|---|
| 1 | 072 | MQIBQAJHZNOHAA-UHFFFAOYSA-N | 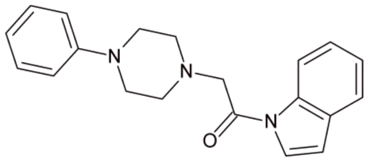 |
| 2 | 102 | SLIKVHAAPXFGRD-UHFFFAOYSA-N | 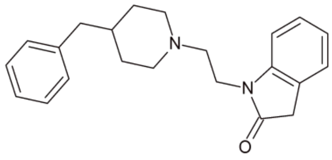 |
| 3 | 032 | GDXUDEPZYCTDRO-UHFFFAOYSA-N | 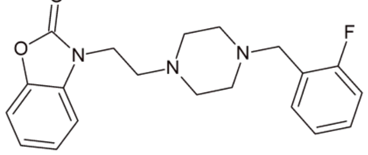 |

| | | | |
|---|---|---|---|
| 4 | 002 | AIVBDSCAWIMJDN-UHFFFAOYSA-N | 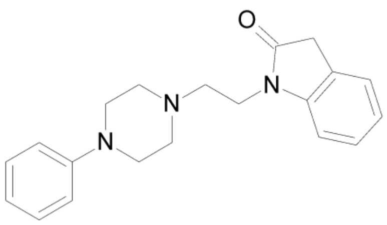 |
| 5 | 016 | CFKMWKYFDWGASU-UHFFFAOYSA-N | 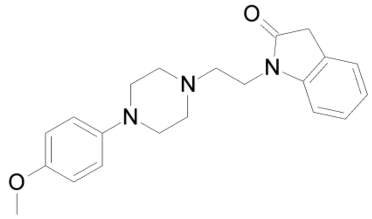 |
| 6 | 041 | HJVFHZSCQNEUTC-UHFFFAOYSA-N | 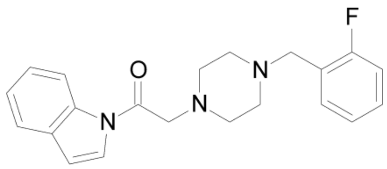 |
| 7 | 042 | HPIGGWPQJAKIDE-UHFFFAOYSA-N | 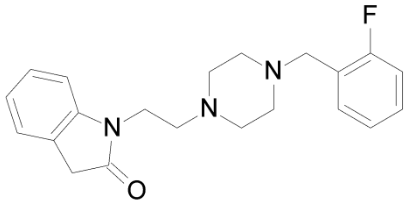 |
| 8 | 078 | NXQBQCLKRHMEPA-UHFFFAOYSA-N | 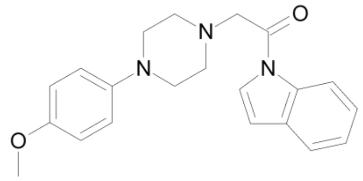 |
| 9 | 090 | QPQKBNDQZHQKRO-UHFFFAOYSA-N | 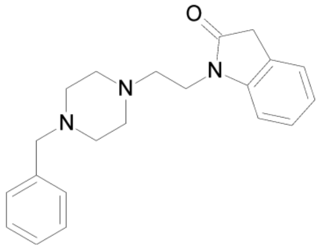 |

| | | | |
|---|---|---|---|
| 10 | 113 | UIVHSJOLXVQEPU-UHFFFAOYSA-N | 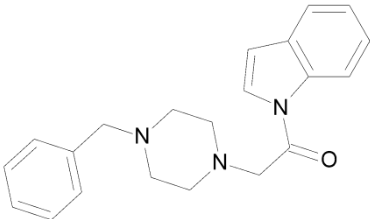 |
| 11 | 086 | PQWODLOHIQGKGB-NTCAYCPXSA-N | 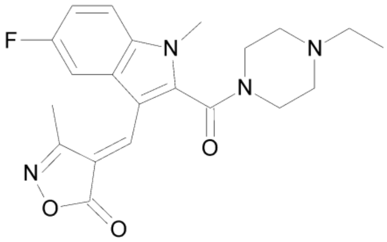 |

**Table S8.** Redocking results of the reference ligand within the Sig-1R receptor using AutoDock Vina. Predicted binding affinity values (kcal·mol$^{-1}$) were used as an additional criterion) are reported for each binding mode, along with the corresponding structural deviations (RMSD l.b. and RMSD u.b.), which represent the minimum and maximum geometric distances of each pose relative to the lowest-energy docking pose, as defined by AutoDock Vina.

| Pose | Affinity (kcal/mol) | RMSD l.b. | RMSD u.b. |
|---|---|---|---|
| 1 | -9.900 | 0.000 | 0.000 |
| 2 | -9.616 | 1.478 | 8.510 |
| 3 | -9.521 | 1.462 | 8.516 |
| 4 | -9.516 | 1.710 | 2.268 |
| 5 | -9.503 | 2.013 | 3.119 |
| 6 | -9.313 | 1.553 | 8.871 |
| 7 | -9.133 | 1.518 | 8.463 |
| 8 | -9.112 | 2.003 | 8.750 |
| 9 | -8.960 | 1.577 | 8.834 |
| 10 | -8.899 | 2.377 | 8.972 |

**Table S9.** Redocking results of the reference ligand within the MAO-B active site using AutoDock Vina. Predicted binding affinity values (kcal·mol$^{-1}$) were used as an additional criterion) are reported for each binding mode, along with the corresponding structural deviations (RMSD l.b. and RMSD u.b.), which represent the minimum and maximum geometric distances of each pose relative to the lowest-energy docking pose, as defined by AutoDock Vina.

| Pose | Affinity (kcal/mol) | RMSD l.b. | RMSD u.b. |
|---|---|---|---|
| 1 | -10.139 | 0 | 0 |
| 2 | -10.049 | 0.509 | 1.112 |
| 3 | -9.872 | 1.247 | 1.866 |
| 4 | -9.866 | 0.979 | 1.385 |
| 5 | -9.822 | 1.109 | 1.754 |
| 6 | -9.772 | 4.814 | 9.451 |
| 7 | -9.679 | 4.452 | 9.189 |
| 8 | -9.615 | 4.868 | 9.609 |
| 9 | -9.248 | 4.84 | 9.235 |

**Table S10.** Virtual screening results for the top 10 ranked ligands targeting the Sigma-1 receptor (Sig-1R) using AutoDock Vina.

| Index | Compound Index | InChIKey | Affinity (kcal/mol) |
|---|---|---|---|
| 01 | 122 | VOCJGRFQDSGYOJ-HDARYGDVSA-N | -12.409 |
| 02 | 138 | YPJYLGAPKFJJMO-HDARYGDVSA-N | -12.182 |
| 03 | 104 | SWXAVUNIIFCCSX-JLUADYMRSA-N | -11.621 |
| 04 | 057 | JXIIMJTUXNELED-IOOJMYGHSA-N | -11.494 |
| 05 | 072 | MQIBQAJHZNOHAA-UHFFFAOYSA-N | -11.395 |
| 06 | 041 | HJVFHZSCQNEUTC-UHFFFAOYSA-N | -11.387 |
| 07 | 102 | SLIKVHAAPXFGRD-UHFFFAOYSA-N | -11.244 |
| 08 | 021 | CVZODOAACNRJNH-UHFFFAOYSA-N | -11.182 |
| 09 | 106 | SXGFSUBZLBBWMV-YDZHTSKRSA-N | -11.096 |
| 10 | 078 | NXQBQCLKRHMEPA-UHFFFAOYSA-N | -11.080 |

**Table S11.** Virtual screening results for the ten top-ranked ligands targeting MAO-B using AutoDock Vina.

| Index | Compound Index | InChIKey | Affinity (kcal/mol) |
|---|---|---|---|
| 1 | 108 | TWQYUHXEMTULPY-VSWUEAEMSA-N | -11.588 |
| 2 | 102 | SLIKVHAAPXFGRD-UHFFFAOYSA-N | -11.307 |
| 3 | 072 | MQIBQAJHZNOHAA-UHFFFAOYSA-N | -11.249 |
| 4 | 104 | SWXAVUNIIFCCSX-JLUADYMRSA-N | -10.418 |
| 5 | 009 | BLGVPQNIUFDMIU-HKOYGPOVSA-N | -10.404 |
| 6 | 002 | AIVBDSCAWIMJDN-UHFFFAOYSA-N | -10.365 |
| 7 | 016 | CFKMWKYFDWGASU-UHFFFAOYSA-N | -10.267 |
| 8 | 032 | GDXUDEPZYCTDRO-UHFFFAOYSA-N | -10.258 |
| 9 | 106 | SXGFSUBZLBBWMV-YDZHTSKRSA-N | -10.233 |
| 10 | 142 | ZCMNPWDYBYIXQC-CSKARUKUSA-N | -10.193 |

**Table S12**. Compounds 102 and 072 showed structurally stable complexes over the simulated timescale, supporting their prioritization as dual-activity potential candidates against MAO-B and Sig-1R.

| Index | Compound Index | InChIKey | Classified structures |
|---|---|---|---|
| 1 | 072 | MQIBQAJHZNOHAA-UHFFFAOYSA-N | 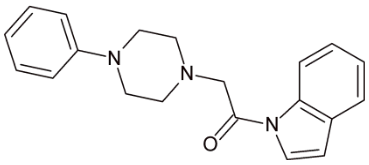 |
| 2 | 102 | SLIKVHAAPXFGRD-UHFFFAOYSA-N | 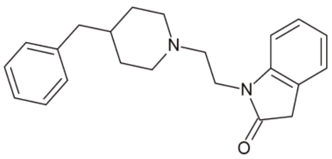 |

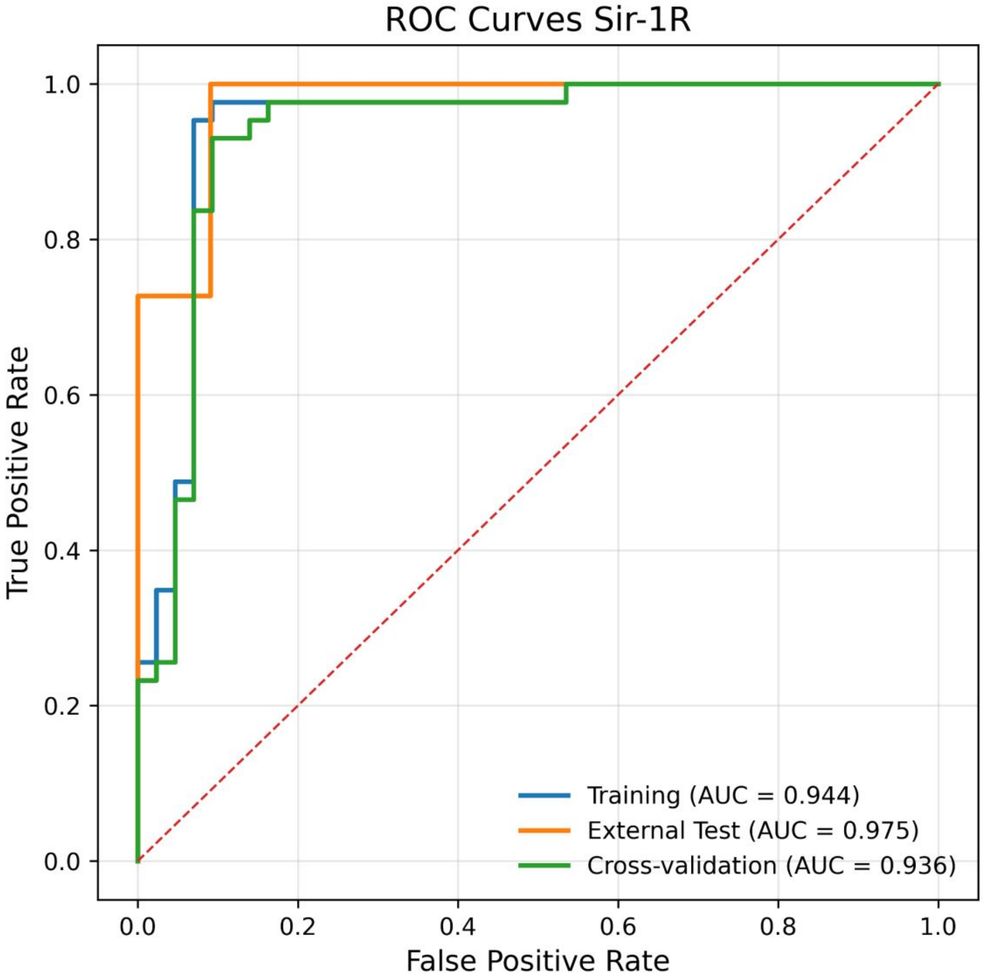


**Figure S1.** Receiver Operating Characteristic (ROC) curve for the Sig-1R model. The graph shows the area under the curve (AUC), which corresponds to the model's performance on the training, test, and cross-validation datasets.

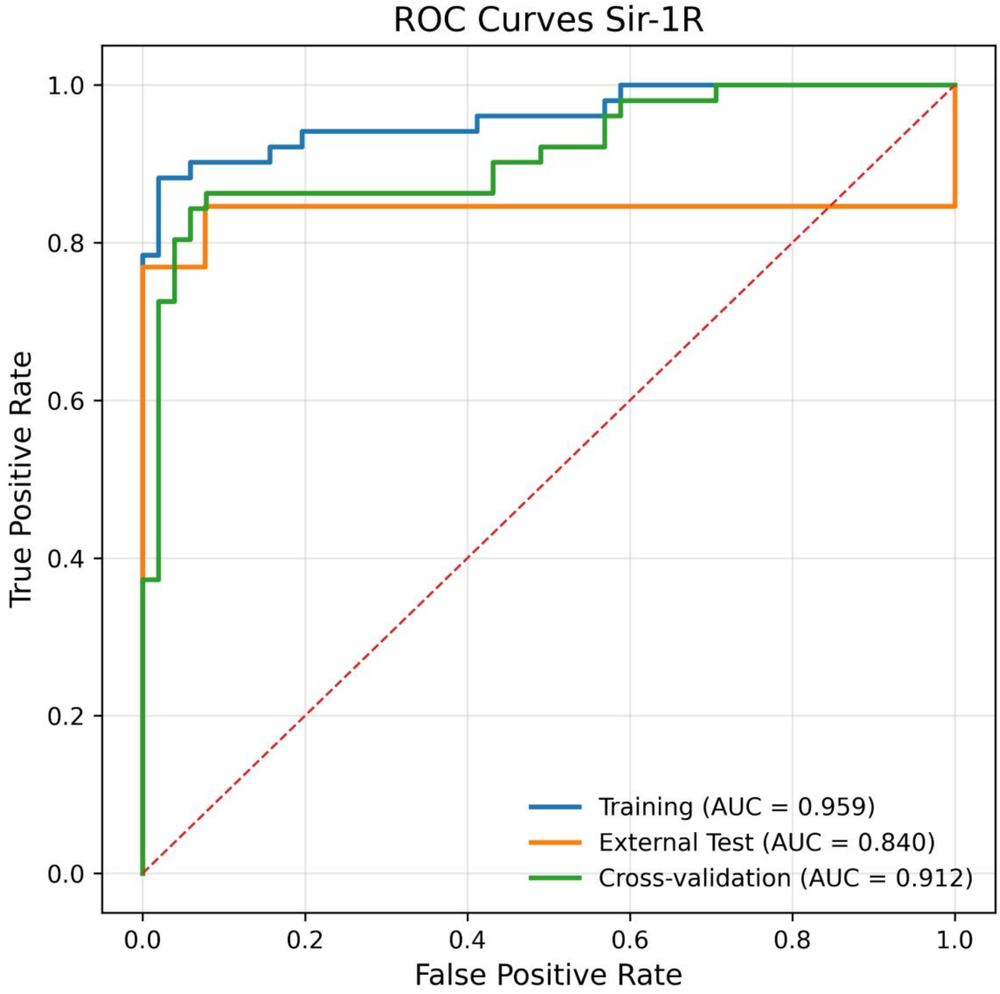


**Figure S2.** Receiver Operating Characteristic (ROC) curve for the MAO-B model. The graph shows the area under the curve (AUC), which corresponds to the model's performance on the training, test, and cross-validation datasets.

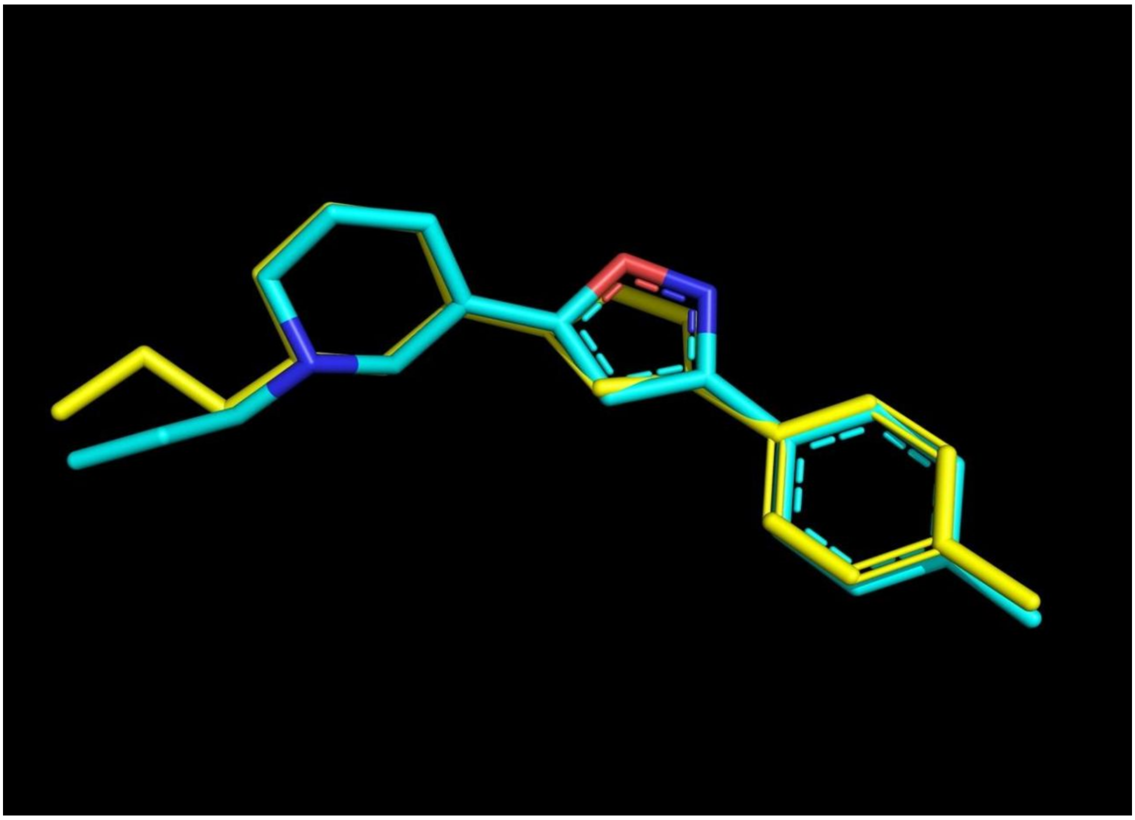

**Figure S3.** Redocking result of the reference ligand PD144418 within the active site of the Sigma-1 receptor (Sig-1R). The lowest-energy pose predicted by AutoDock Vina is shown in yellow, while the crystallographic reference ligand is displayed for structural comparison, yielding an RMSD of 1.478 Å.

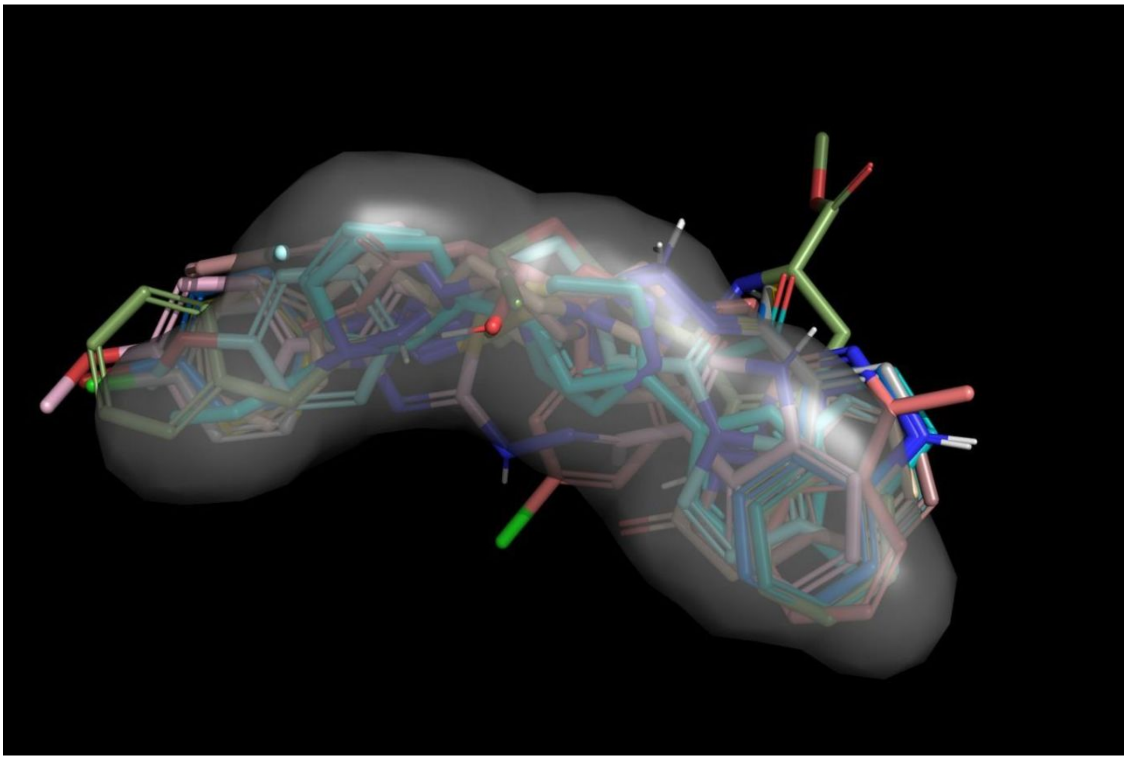

**Figure S4.** Superposition of the docking poses of the ten top-ranked ligands selected by virtual screening within the active site of the Sig-1R receptor.

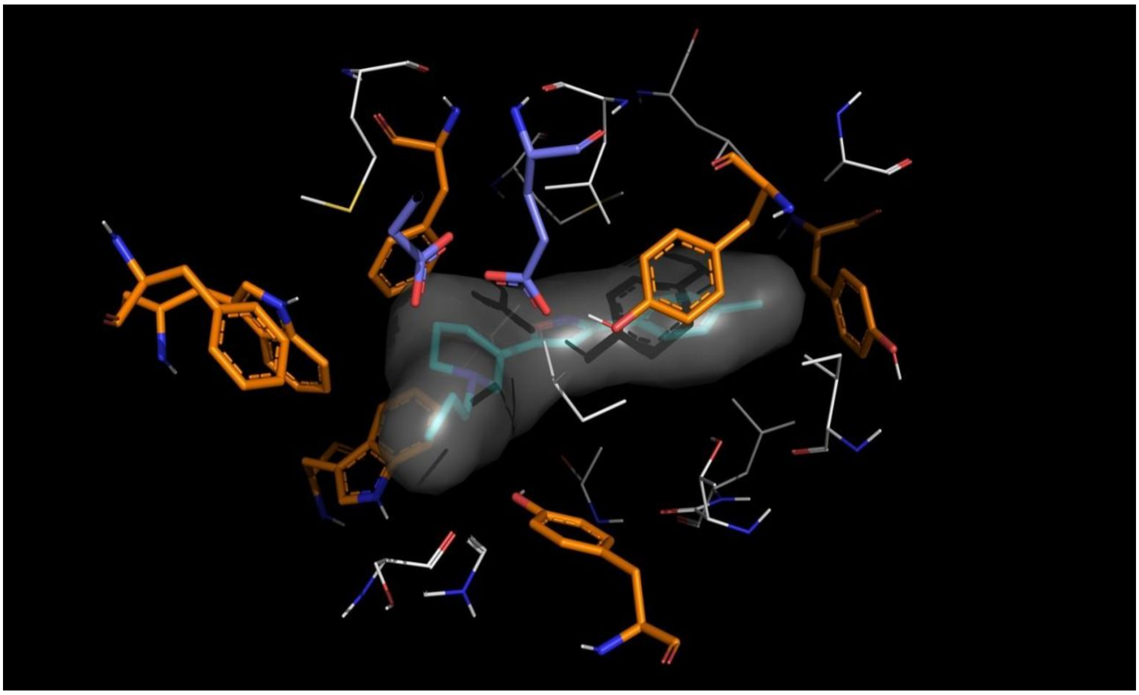

**Figure S5.** Active site of the Sig-1R receptor highlighting the main residues involved in ligand recognition.

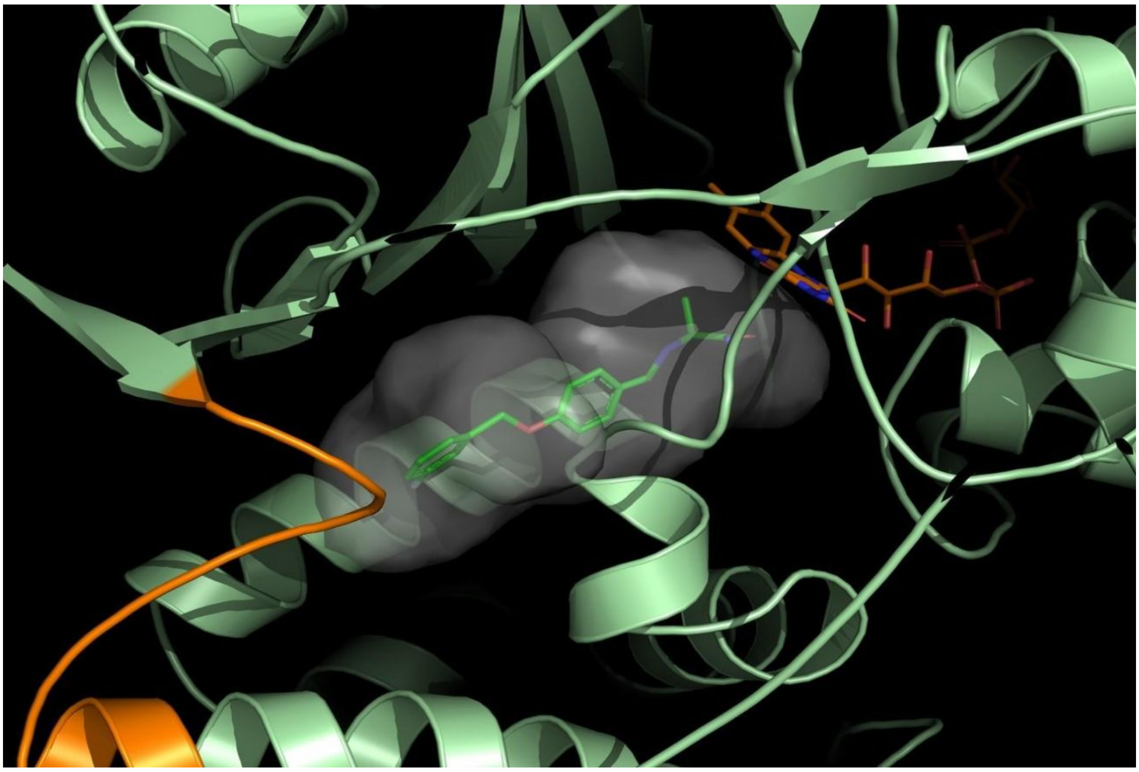

**Figure S6.** Representation of the binding mode of safinamide within the active site of monoamine oxidase B (MAO-B). The ligand (shown in green) is fitted within the catalytic channel, occupying

the hydrophobic cavity adjacent to the FAD cofactor (shown in orange, right) and interacting with the MAO-B selectivity loop (shown in orange, left), which contributes to the differentiation between the MAO-A and MAO-B isoforms and is associated with the selective recognition of inhibitors.

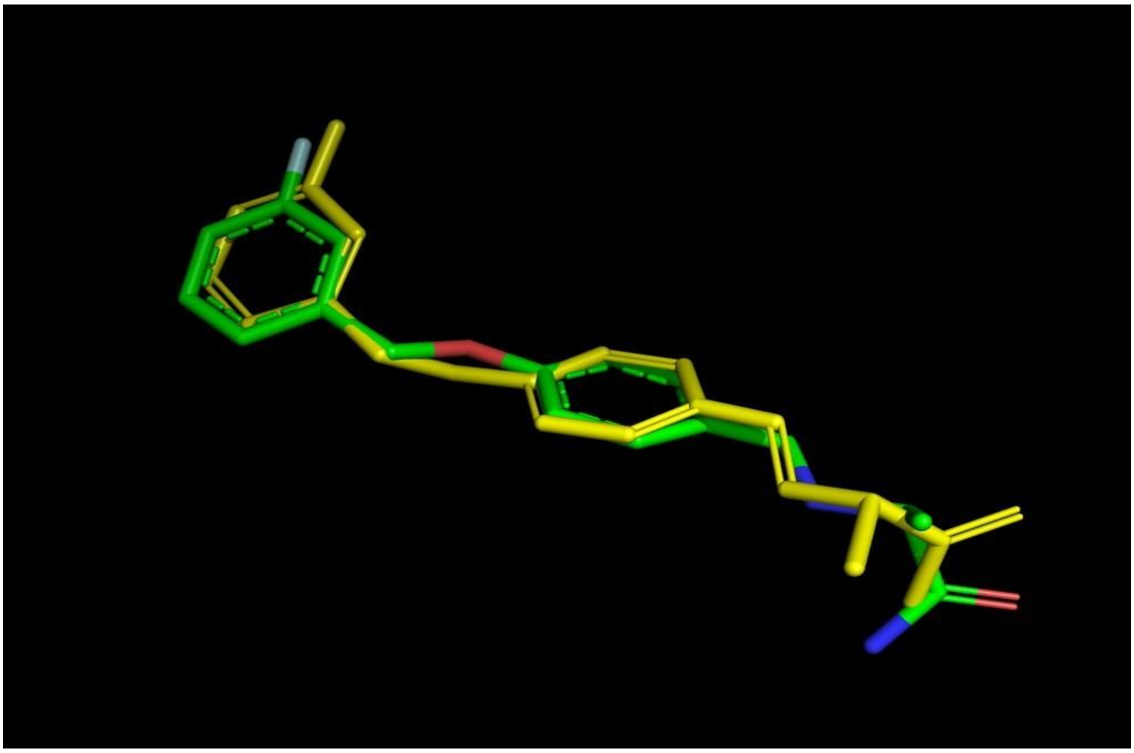

**Figure S7.** Redocking result of the reference ligand safinamide within the active site of monoamine oxidase B (MAO-B). The lowest-energy pose predicted by AutoDock Vina is shown in yellow, while the crystallographic reference ligand is displayed for structural comparison. Structural alignment of the two conformations yielded an RMSD value of 0.509 Å, indicating accurate reproduction of the experimental binding mode.

**Figure S8.** Superposition of the ten best docking compounds retrieved from virtual screening within the active site of monoamine oxidase B (MAO-B). The different ligand conformations (represented in distinct colours) highlight the preferential occupation of the hydrophobic catalytic channel, showing good steric complementarity with the active site cavity (surface shown in grey).

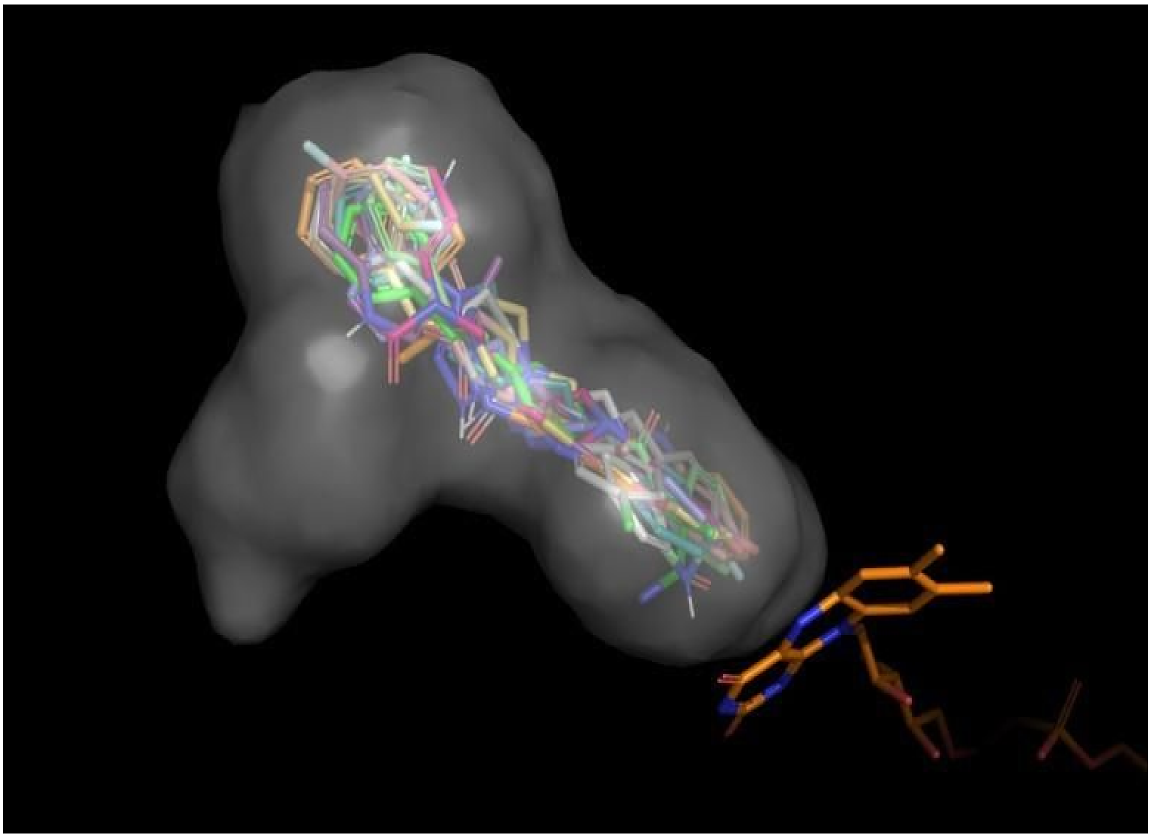

The FAD cofactor (shown in orange) is displayed for spatial reference, emphasising the proximity of the poses to the catalytic region of the enzyme.

**Figure S9.** RMSD of the MAO-B protein complexed with different ligands during 150 ns. For clarity, the RMSD values in Figure S9 are reported in Å; thus, values above 6 Å correspond to approximately 0.6 nm and should not be interpreted as 0.6 Å.

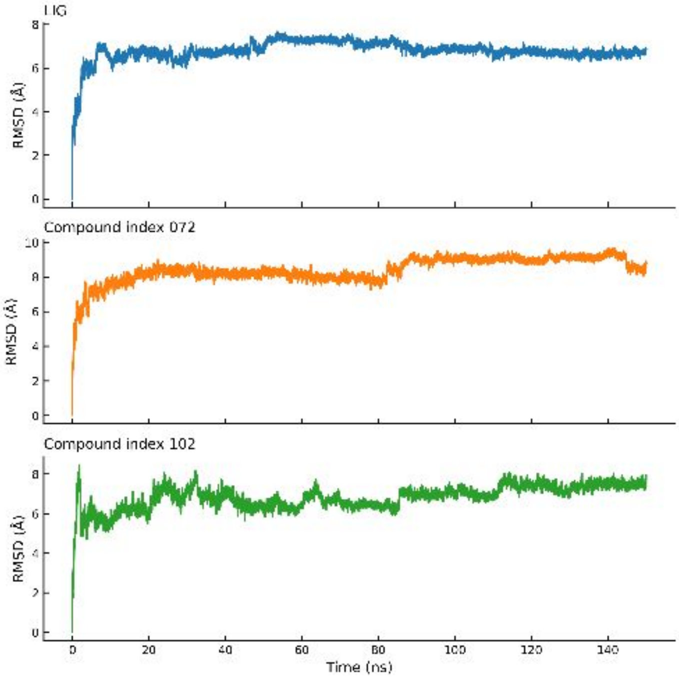

**Figure S10.** RMSD of the Sig-1R protein complexed with different ligands during 150 ns.

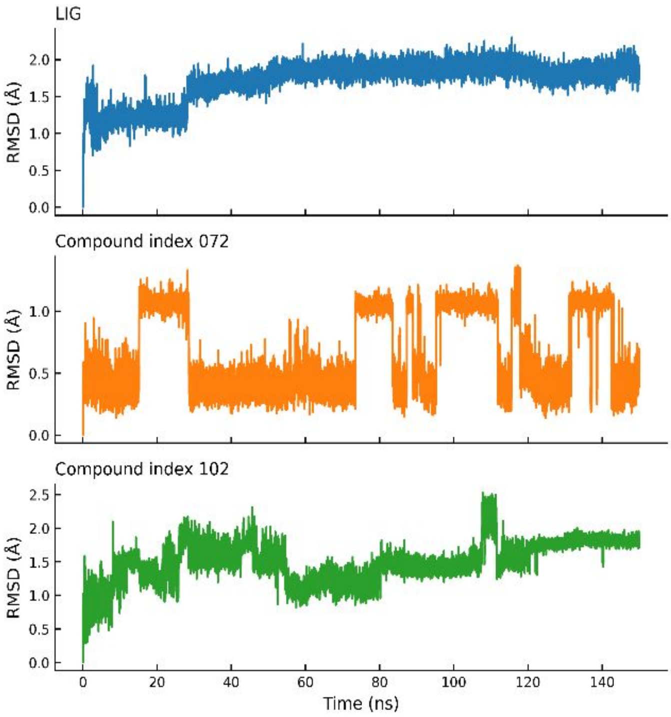

**Figure S11**. Ligand RMSD during 150 ns of simulation within the MAO-B protein.

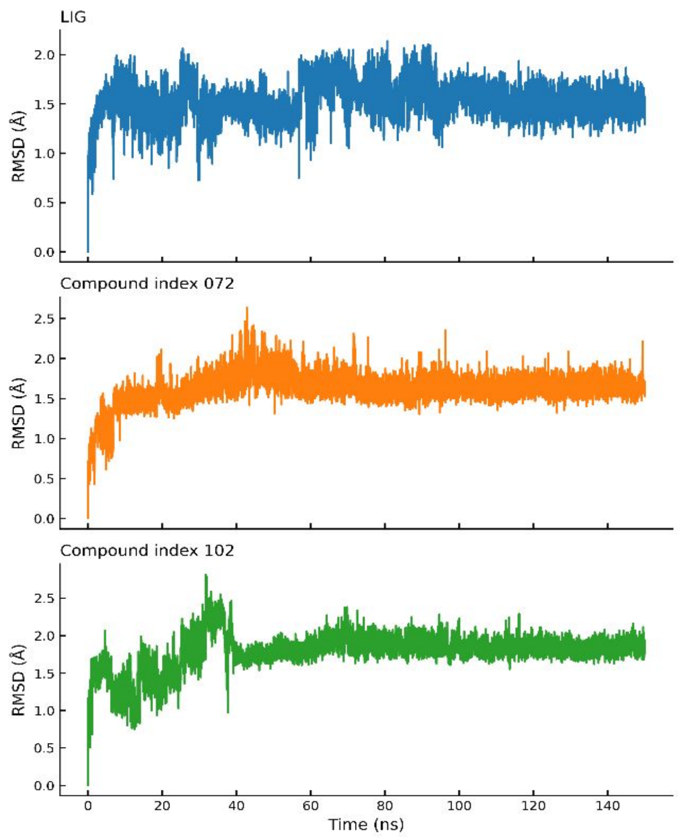


**Figure S12.** Ligand RMSD during 150 ns of simulation within the Sig-1R protein.

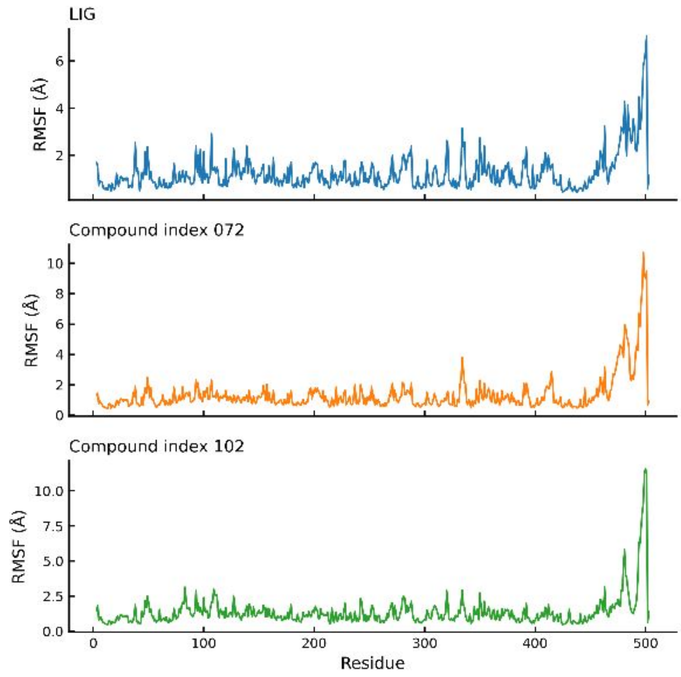

**Figure S13.** RMSF of the MAO-B protein complexed with different ligands during 150 ns.

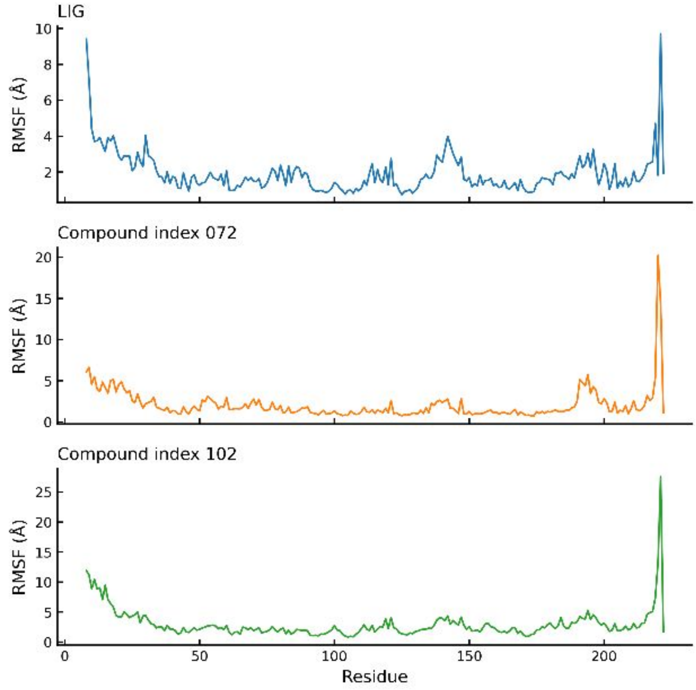


**Figure S14.** RMSF of the Sig-1R protein complexed with different ligands during 150 ns.

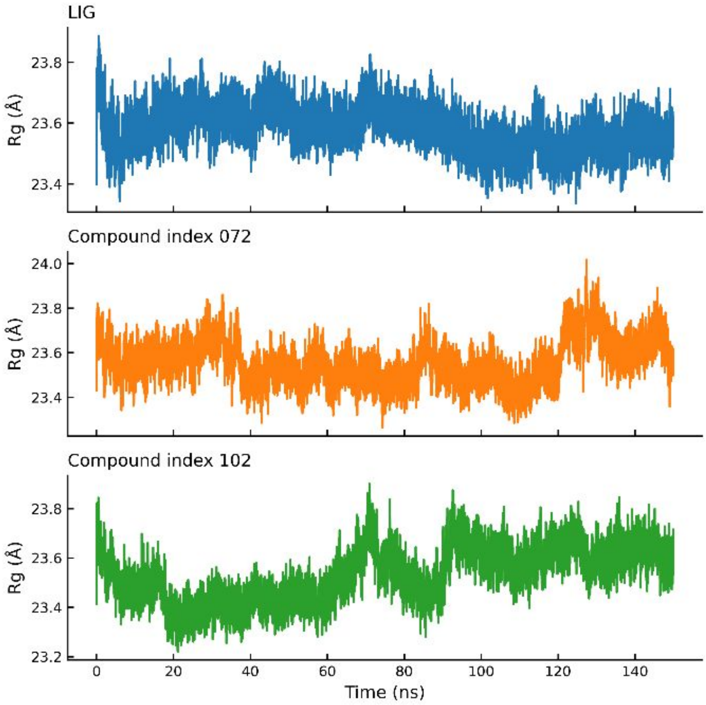

**Figure S15.** Radius of Gyration ($R_g$) of the MAO-B protein complexed with different ligands during 150 ns.

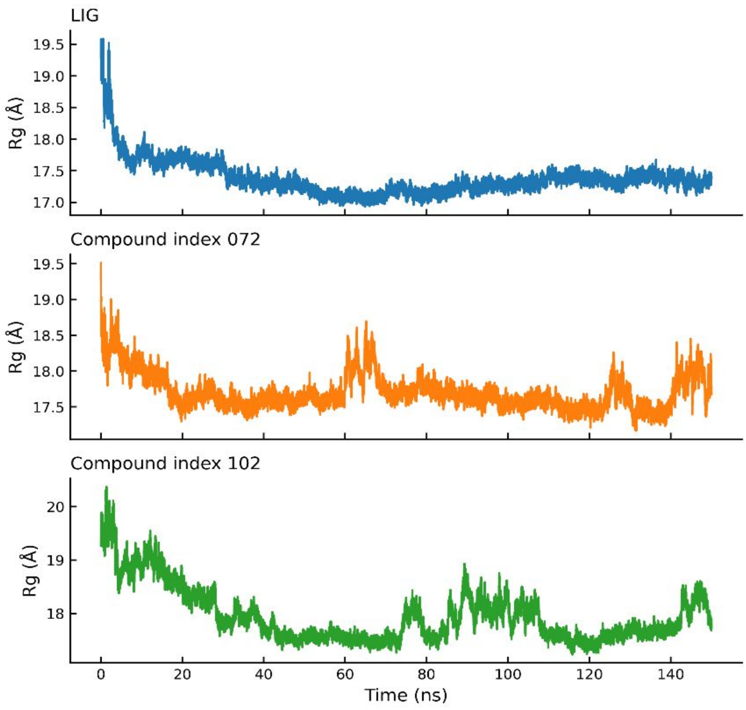


**Figure S16.** Radius of Gyration ($R_g$) of the Sig-1R protein complexed with different ligands during 150 ns.

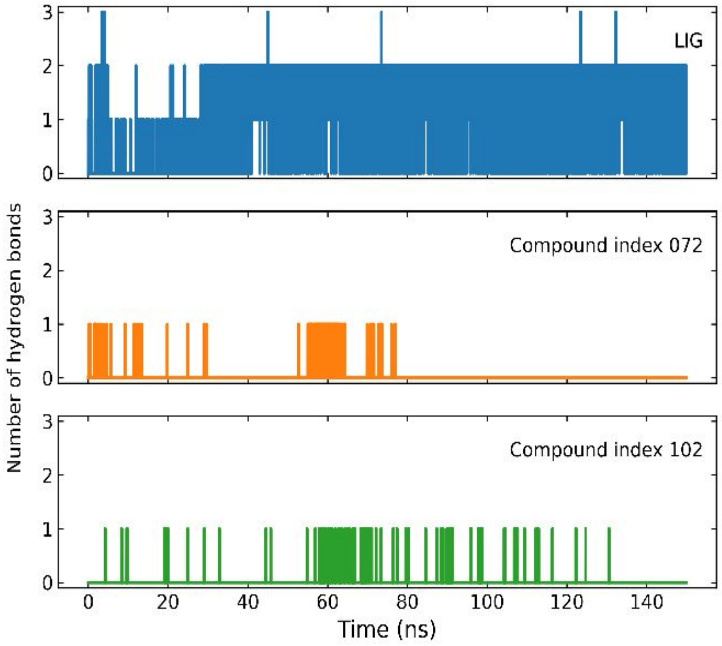

**Figure S17.** Number of H-bonds in the MAO-B protein with different ligands during 150 ns.

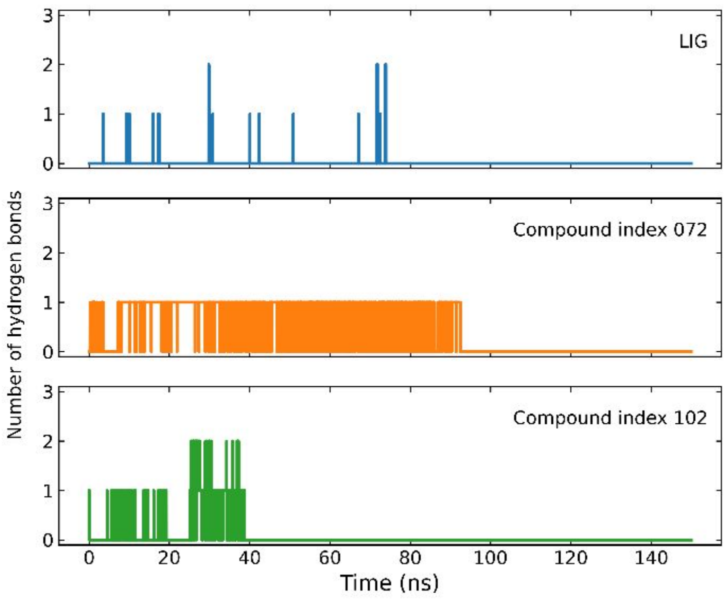


**Figure S18.** Number of H-bonds in the Sig-1R protein with different ligands during 150 ns.

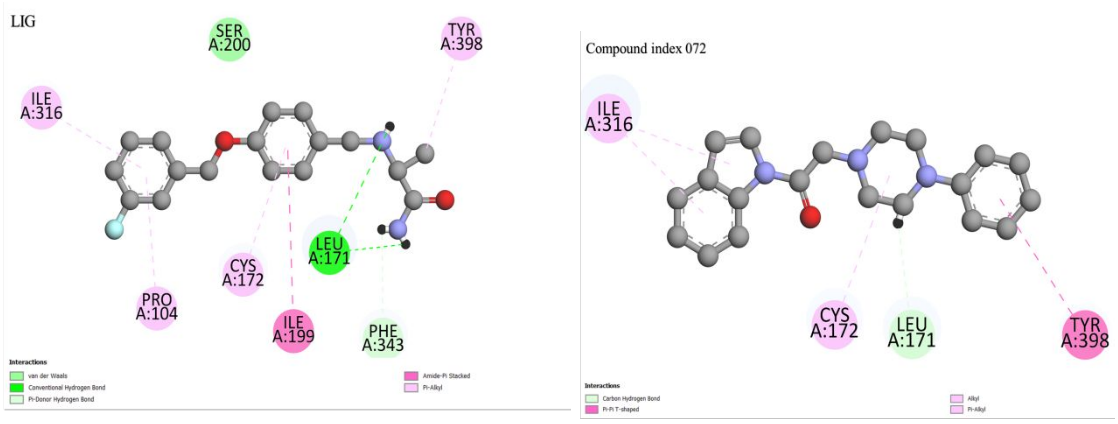

**Figure S19**. Interactions for the most representative complexes during the molecular dynamics simulation for the MAO-B target.

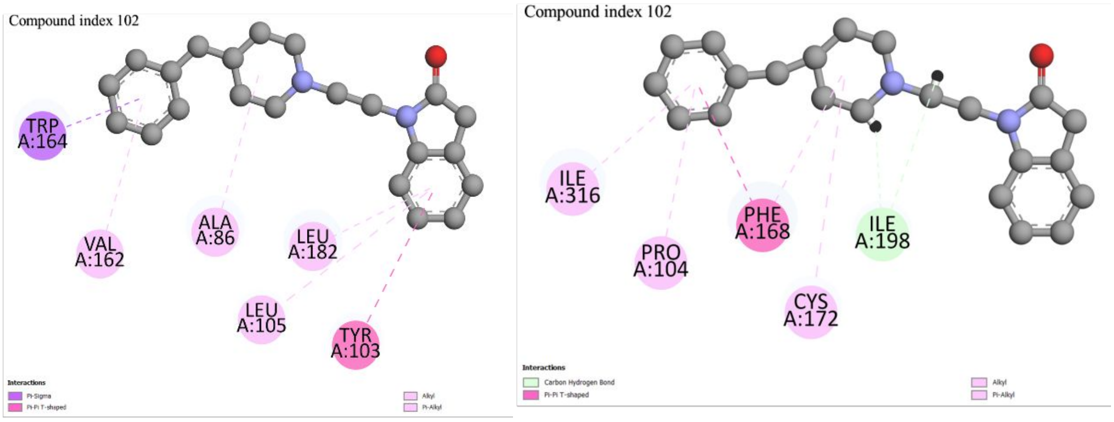


**Figure S20**. Interactions for the most representative complexes during the molecular dynamics simulation for the Sig-1R target.

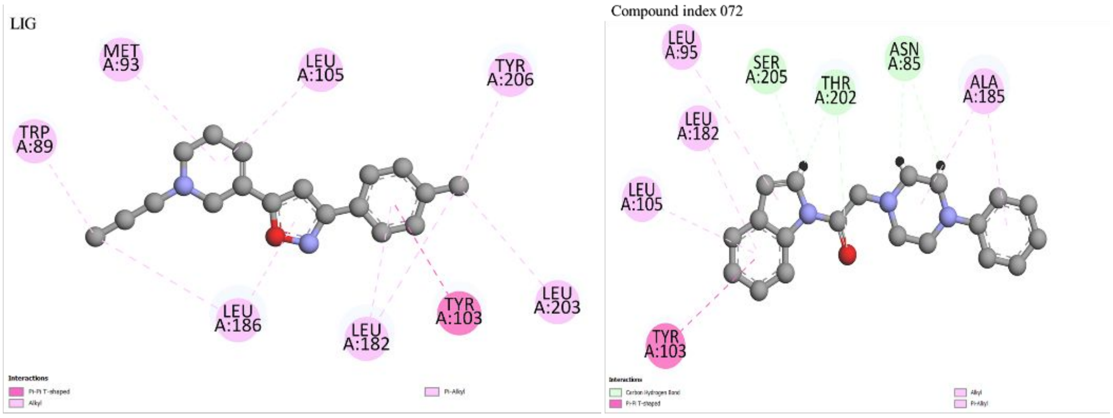


## Supplementary structural validation of the docking protocol by redocking and cross-docking

To further support the structural consistency of the docking protocol, additional redocking and cross-docking experiments were carried out for Sig1R and MAO-B using crystallographic ligands from independent PDB complexes. This analysis was designed to assess whether the protocol could both reproduce known crystallographic poses and accommodate additional ligands in the receptor structures used in the main study.

## Sig-1R

### Redocking of the crystallographic ligand from 5HK2

The crystallographic ligand from the 5HK2 complex was first redocked into its native receptor structure. The best-ranked pose showed a predicted affinity of -10.12 kcal/mol. RMSD analysis indicated excellent pose recovery, with the first two poses showing RMSD values below 1 Å relative to the crystallographic reference (0.6080 Å and 0.6150 Å, respectively). These results support the ability of the protocol to reproduce the experimental binding mode of the 5HK2 ligand

**Table S13.** AutoDock Vina results for redocking of the 5HK2 crystallographic ligand into its native Sig-1R receptor.

| Pose | Affinity (kcal/mol) | RMSD l.b. (Å) | RMSD u.b. (Å) |
|---|---|---|---|
| 1 | -10.12 | 0 | 0 |
| 2 | -10.10 | 0.03179 | 1.004 |
| 3 | -9.884 | 3.541 | 9.288 |
| 4 | -9.701 | 3.467 | 9.280 |
| 5 | -9.684 | 3.459 | 9.317 |
| 6 | -9.592 | 3.561 | 9.386 |
| 7 | -9.591 | 1.260 | 2.031 |
| 8 | -9.076 | 3.561 | 9.403 |
| 9 | -8.820 | 3.601 | 8.956 |
| 10 | -8.612 | 3.737 | 9.613 |

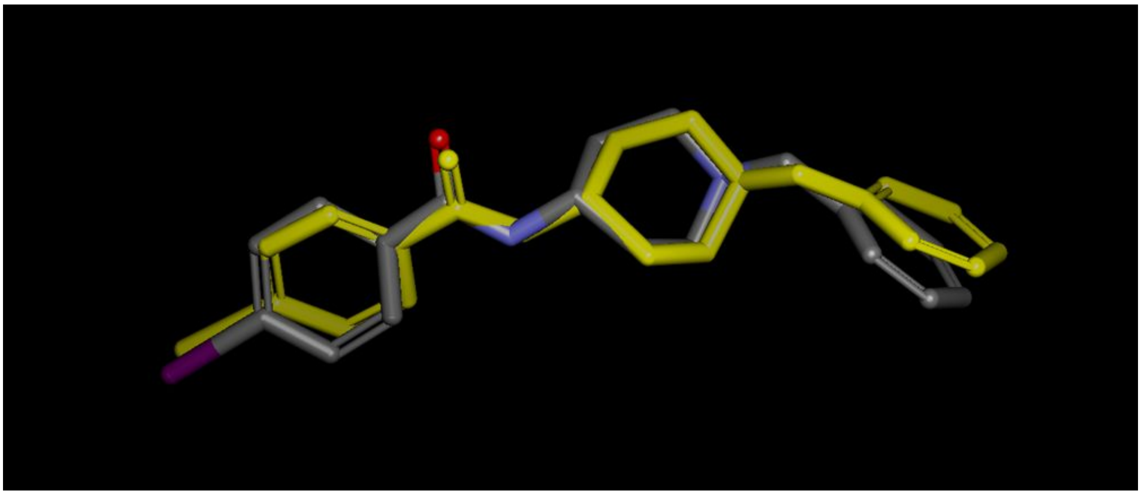

**Figure S21.** Superposition of the crystallographic pose of the 5HK2 ligand and the best redocked pose.

**Cross-docking of the 5HK2 ligand into 5HK1**

The crystallographic ligand from **5HK2** was then docked into the **5HK1** receptor, which was used as the main Sig1R structure in this study. The top-ranked poses showed favorable predicted affinities, with the first four poses ranging from **-10.61 to -10.55 kcal/mol**. Visual inspection indicated that the ligand was placed in the same binding region and adopted an orientation consistent with the known ligand-binding environment of Sig-1R. These results provide additional structural support for the robustness of the docking setup

**Table S14.** AutoDock Vina results for cross-docking of the 5HK2 crystallographic ligand into the 5HK1 Sig-1R receptor.

| Pose | Affinity (kcal/mol) | RMSD l.b. ( Å) | RMSD u.b. ( Å) |
|---|---|---|---|
| 1 | -10.61 | 0 | 0 |
| 2 | -10.61 | 0.04151 | 1.004 |
| 3 | -10.59 | 0.1619 | 1.428 |
| 4 | -10.55 | 0.1297 | 1.011 |
| 5 | -9.811 | 3.852 | 9.350 |
| 6 | -9.796 | 3.856 | 9.406 |
| 7 | -9.711 | 1.088 | 1.857 |
| 8 | -9.587 | 1.892 | 2.932 |
| 9 | -9.470 | 1.921 | 2.558 |
| 10 | -8.974 | 3.807 | 9.779 |

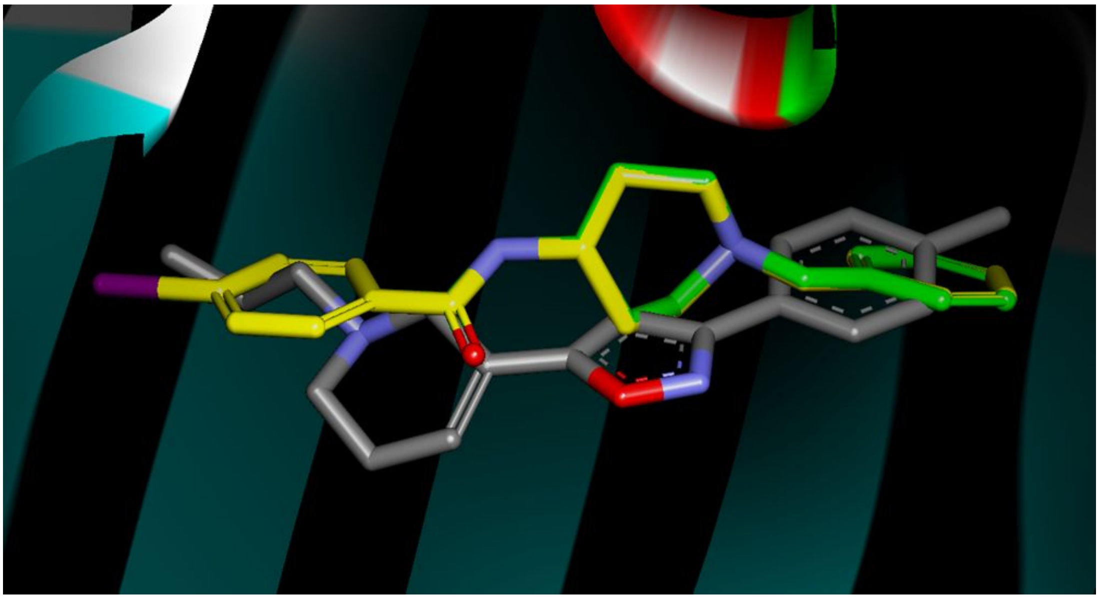

**Figure S22.** Cross-docking of the crystallographic ligand from 5HK2 into 5HK1.

## MAO-B

### Redocking of the crystallographic ligand from 2V61

For MAO-B, the crystallographic ligand from 2V61 was first redocked into its native receptor structure. The best-ranked pose showed a predicted affinity of -10.29 kcal/mol. RMSD analysis showed that poses 1 and 2 reproduced the crystallographic conformation with values below 2 Å (1.1288 Å and 1.7870 Å, respectively), indicating satisfactory recovery of the experimental binding mode

**Table S15.** AutoDock Vina results for redocking of the 2V61 crystallographic ligand into its native MAO-B receptor.

| Pose | Affinity (kcal/mol) | RMSD l.b. (Å) | RMSD u.b. (Å) |
|---|---|---|---|
| 1 | -10.29 | 0 | 0 |
| 2 | -10.20 | 0.8734 | 1.342 |
| 3 | -9.939 | 4.006 | 8.441 |
| 4 | -9.939 | 4.075 | 8.492 |
| 5 | -9.922 | 3.787 | 8.268 |
| 6 | -9.815 | 1.776 | 2.426 |
| 7 | -9.746 | 3.770 | 8.408 |
| 8 | -9.742 | 1.985 | 2.776 |
| 9 | -9.716 | 1.364 | 1.925 |

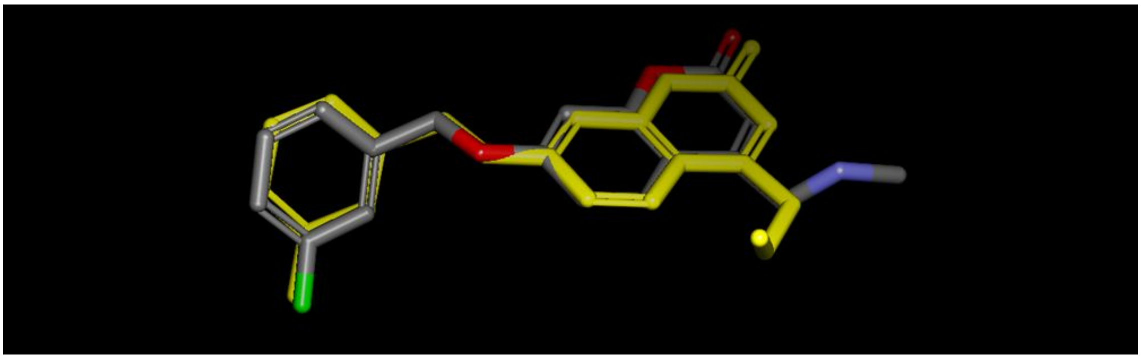

**Figure S23.** Superposition of the crystallographic pose of the 2V61 ligand and the best redocked pose.

### Cross-docking of the 2V61 ligand into 2V5Z

The crystallographic ligand from 2V61 was subsequently docked into the 2V5Z receptor, which was the MAO-B structure used in the main study. The best-ranked pose showed a predicted affinity of -10.46 kcal/mol. Visual inspection indicated that the ligand occupied the same active-site region and adopted an orientation consistent with the binding

environment observed for safinamide in the 2V5Z complex. Together, these results indicate that the receptor used in the study was also able to accommodate a crystallographic ligand from an independent MAO-B complex in a structurally coherent manner.

**Table S16.** AutoDock Vina results for cross-docking of the 2V61 crystallographic ligand into the 2V5Z MAO-B receptor.

| Pose | Affinity (kcal/mol) | RMSD l.b. (Å) | RMSD u.b. (Å) |
|---|---|---|---|
| 1 | -10.46 | 0 | 0 |
| 2 | -10.38 | 0.8667 | 1.335 |
| 3 | -10.30 | 2.052 | 2.841 |
| 4 | -10.24 | 1.871 | 2.502 |
| 5 | -10.21 | 4.271 | 8.588 |
| 6 | -10.09 | 1.341 | 1.621 |
| 7 | -9.977 | 3.909 | 8.283 |
| 8 | -9.852 | 4.164 | 8.526 |
| 9 | -9.513 | 2.130 | 3.131 |
| 10 | -9.032 | 2.574 | 3.978 |

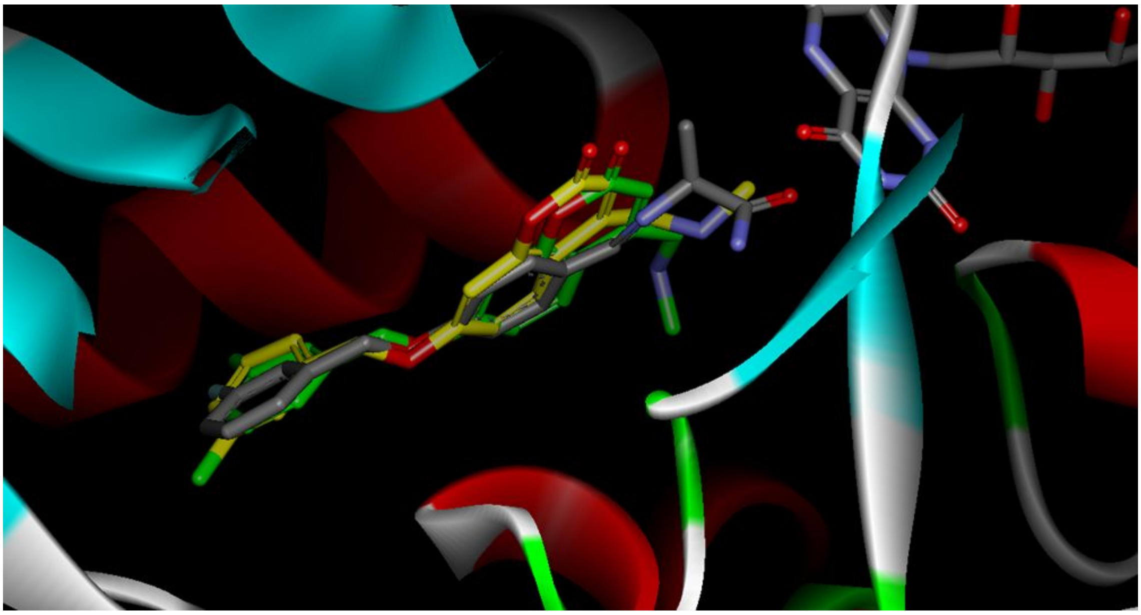

**Figure S24.** Cross-docking of the crystallographic ligand from 2V61 into 2V5Z.
